# Microfluidic-Integrated CRISPR-Cas Biosensor for Marine Pollutant and Pathogen Monitoring: A Review

**DOI:** 10.3390/bios16090483

**Published:** 2026-09-01

**Authors:** Natalia Binti Ali, Yuting Xiao, Kuiyu Jin, Yixuan Wang, Youquan Zhao

**Affiliations:** 1 Medical School, Tianjin University, Tianjin 300072, China; 2024246004@tju.edu.cn (Y.X.); jky2001@tju.edu.cn (K.J.); 2024246099@tju.edu.cn (Y.W.); 2 School of Precision Instrument and Opto-Electronics Engineering, Tianjin University, Tianjin 300072, China

**Keywords:** CRISPR-Cas12a/Cas13a, lab-on-a-chip, point-of-care diagnostics, harmful algal bloom toxins, heavy metal ion detection, antibiotic resistance genes, isothermal amplification, seawater analysis

## Abstract

Marine ecosystems face escalating threats from heavy metals, harmful algal bloom toxins, pathogens, and antibiotic resistance genes, yet conventional detection methods remain laboratory-dependent and incapable of real-time, multiplexed field monitoring. CRISPR-Cas diagnostics, leveraging programmable Cas12a/Cas13a *trans*-cleavage for attomolar-level sensitivity, offers a transformative solution when integrated with microfluidic platforms that provide the automation and miniaturisation required for field deployment. This review systematically examines this emerging convergence across four marine target classes: heavy metals, biotoxins, pathogens, and resistance genes alongside integration architectures, signal readout strategies, and comparative performance benchmarking. We identify that only a small fraction of reported platforms have been validated in authentic seawater, with cross-class multiplexing, biofouling resistance during autonomous deployment, and regulatory standardisation remaining largely unaddressed. By synthesising this rapidly developing literature and articulating these unresolved challenges, this review provides a foundational reference and research agenda for translating microfluidic-CRISPR biosensors from laboratory proof-of-concept to operational marine environmental surveillance.

## 1. Introduction

Marine ecosystems face escalating contamination from four major pollutant classes: heavy metals, harmful algal bloom (HAB) toxins, pathogenic microorganisms, and antibiotic resistance genes (ARGs)—collectively threatening marine biodiversity and seafood safety [1]. Heavy metals such as cadmium, lead, and mercury bioaccumulate through the marine food web causing reproductive impairment and acute toxicity, while HAB events documented at 9503 occurrences across nine ocean regions since 1985 [2]—can cause single-event losses exceeding USD 800 million [3]. The ocean has further become a critical reservoir for antimicrobial resistance, with terrestrial ARGs disseminating into marine ecosystems via horizontal gene transfer [4], compounding pressures from nutrient-driven dead zones now spanning thousands of square miles [5]. These converging threats underscore the urgent need for sensitive, field-deployable detection platforms.

Conventional analytical methods like ICP-MS, HPLC, culture-based assays, and optical biosensors remain laboratory-dependent, single-target, and largely unvalidated in authentic marine matrices [6,7,8]. No existing platform addresses the simultaneous, multiplexed detection of chemically diverse marine threats within a single deployable system [9]—a gap that CRISPR-Cas diagnostics, now at a defining stage of technological maturity, are positioned to address.

CRISPR-Cas diagnostics has progressed through clear technological milestones directly relevant to this gap. At the mechanistic level, the programmable *trans*-cleavage ability of Type V and VI CRISPR-Cas nucleases (particularly Cas12a and Cas13a) underpins their exceptional utility in biosensing, enabling attomolar-level sensitivity through collateral cleavage of fluorescent reporter probes upon target recognition [10]. Unlike PCR-based methods, CRISPR reactions operate at physiological temperature, making them inherently compatible with point-of-care assembly, while microfluidic integration further enables sample processing and analysis on a single chip with reduced reagent consumption and increased throughput [11,12]—a synergy particularly suited to the marine context, where microfluidic automation directly addresses the complex matrix effects that have historically prevented biosensor translation to open-ocean environments [12]. Most recently, a field-deployable CRISPR biosensing platform demonstrated versatility across three climate-linked marine indicators—*Vibrio* spp., *Pseudo-nitzschia* spp., and heat-stressed corals—using portable 3D-printed devices for direct field sample processing [13], confirming the field-applicability of this convergence is no longer theoretical. Despite this momentum, existing reviews address CRISPR-based diagnostics and marine environmental biosensing as separate studies, each considering at most one or two target classes in isolation. To our knowledge, this review is the first to integrate microfluidic platforms, CRISPR-Cas effectors, and marine-specific pollutant and pathogen detection—spanning heavy metals, biotoxins, ARGs, and pathogens within a unified analytical framework.

**Figure 1:**
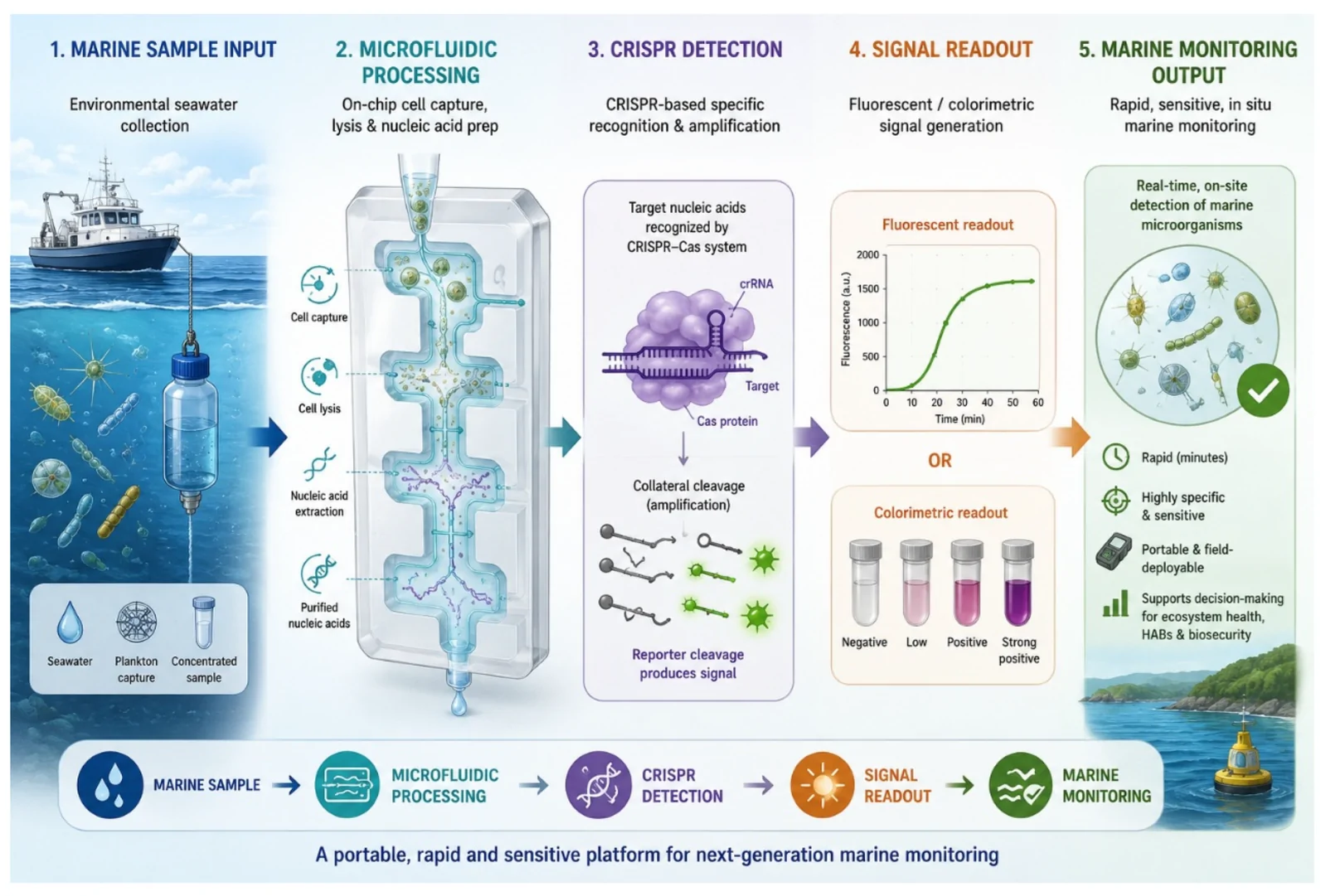
Overview of the microfluidic-CRISPR marine biosensing workflow. Seawater or plankton-concentrated samples undergo on-chip cell capture, lysis, and nucleic acid extraction; the purified nucleic acids are recognized by a CRISPR–Cas effector, triggering collateral (*trans*-) cleavage of quenched reporter probes; the resulting fluorescent or colorimetric signal is read out to support rapid, sensitive, portable, in situ monitoring of marine microorganisms and pollutants. In the colorimetric panel, color intensity from colorless to deep purple corresponds to increasing target concentration/signal strength.

This review provides a comprehensive analysis of this emerging convergence, following the literature search methodology outlined in Section Literature Search Methodology. Following CRISPR-Cas mechanisms and microfluidic architectures (Section 2), we examine integration strategies (Section 3), marine-specific applications (Section 4), signal readout modalities (Section 5), performance benchmarking (Section 6), and persisting challenges and future directions (Section 7). Figure 1 provides a schematic overview of this end-to—end microfluidic-CRISPR marine biosensing workflow.

### Literature Search Methodology

A structured literature search was conducted across Web of Science, PubMed, and Scopus using the terms “CRISPR,” “microfluidics,” “marine detection,” “water quality biosensor,” “heavy metals,” “harmful algal blooms,” “antibiotic resistance genes,” and “marine pathogens,” covering publications from 2018 to 2025. Inclusion criteria comprised peer-reviewed, English-language primary research and review articles reporting a CRISPR-Cas-based (Cas9, Cas12, Cas13, or Cas14) detection or biosensing platform, with or without microfluidic integration, applied to at least one of the four marine-relevant target classes considered here: heavy metals, harmful algal bloom biotoxins or associated eDNA/eRNA, marine or aquaculture pathogens, and antibiotic resistance genes. Studies validated exclusively in freshwater, wastewater, or clinical matrices were retained where the underlying detection chemistry was judged transferable to marine applications, consistent with this review’s objective of mapping the gap between laboratory performance and marine field readiness. Exclusion criteria comprised non-English-language articles, conference abstracts and preprints without peer review, studies addressing CRISPR solely as a genome-editing tool without a biosensing or diagnostic application, and duplicate or lower-performing platform reports superseded by a more comprehensive account from the same research group. Screening of titles, abstracts, and full texts against these criteria yielded the 96 references synthesised across Section 2, Section 3, Section 4, Section 5, Section 6 and Section 7.

## 2. Background and Fundamentals

### 2.1. CRISPR-Cas Effector Mechanisms Relevant to Biosensing

The CRISPR-Cas system comprises a ribonucleoprotein complex in which a Cas nuclease is guided by a programmable CRISPR RNA (crRNA) to recognize and cleave specific nucleic acid sequences [14]. For biosensing applications, two effectors dominate the literature: Cas12a (Type V) and Cas13a (Type VI) [14]. Upon target recognition, Cas12a first undergoes PAM-dependent crRNA invasion into double-stranded DNA [15]. PAM recognition promotes dsDNA unwinding, allowing the unwound target strand to hybridize with the seed region of the crRNA to form an R-loop structure [15]. This induces a conformational change in which the REC lobe moves away from the RuvC domain, exposing the catalytic site [15]. Active Cas12a then executes *cis*-cleavage of the target DNA, after which the ribonucleoprotein remains catalytically competent and indiscriminately cleaves any proximal single-stranded DNA reporters—a process termed *trans*-cleavage [15].

Cas13a operates analogously but targets single-stranded RNA rather than DNA [14,15]. Upon binding its RNA target, Cas13a activates both sequence-specific *cis*-cleavage and collateral *trans*-cleavage of proximal ssRNA reporters via its two HEPN catalytic domains [15,16]. While Cas13a bypasses the need for PAM sequences entirely, some orthologs depend on a Protospacer Flanking Sequence (PFS); however, it is notable that highly active variants utilized in biosensing, such as LwaCas13a, are completely PFS-independent [16]. Emerging effectors such as Cas14 offer PAM-independent ssDNA recognition and have demonstrated superior sensitivity compared to Cas12a in aptasensor configurations, broadening the range of non-nucleic acid marine targets accessible to CRISPR-based detection [17]. Representative demonstrations illustrate this versatility: a Cas14a-porphyrin metal–organic framework sensor achieved a limit of detection of 19 pg/mL (≈19 pM) for the algal toxin microcystin-LR by coupling aptamer-mediated competitive binding to Cas14a *trans*-cleavage [18], directly complementing the microcystin-LR case studies discussed in Section 4.3; and a PAM-free Cas14a1 biosensor combining single-primer isothermal amplification with strand-displacement activation achieved 5 CFU/mL sensitivity for *Salmonella typhi* without requiring a protospacer adjacent motif [19], illustrating the target-recognition flexibility that PAM-independence affords. The same aptasensor architecture has additionally been extended to Cd^2+^, histamine, and aflatoxin B1, suggesting applicability to the heavy-metal and toxin classes reviewed in Section 4.1 and Section 4.3; however, none of these Cas14 platforms has yet been validated in seawater or another authentic marine matrix.

This *trans*-cleavage amplification mechanism—shared by both effectors—is the cornerstone of CRISPR biosensing, underpinning the ultrasensitive detection of both nucleic acid and non-nucleic acid targets [14]. Attomolar-level sensitivity is achievable through the collateral cleavage of fluorophore-quencher reporter probes, which generate easily quantifiable signals [15,20]. Importantly, without a pre-amplification step, CRISPR biosensors typically exhibit detection capabilities restricted to the nM–pM range [21]. Therefore, coupling these systems with isothermal amplification strategies—such as recombinase polymerase amplification (RPA) or loop-mediated isothermal amplification (LAMP)—is commonly required to achieve the attomolar (aM) sensitivity demanded by field-deployable environmental and marine diagnostics [21].

**Figure 2:**
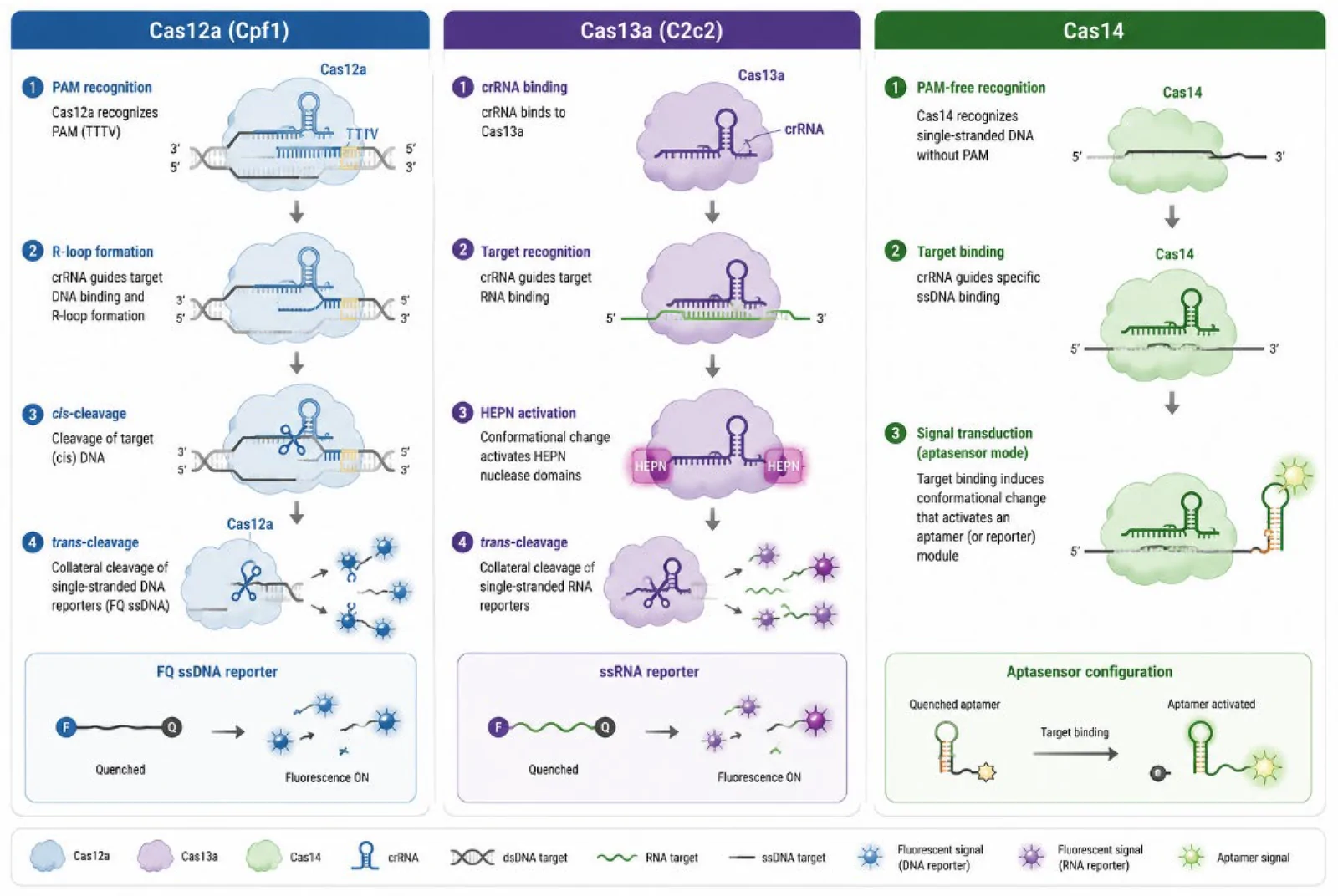
Mechanistic comparison of the three CRISPR-Cas effectors used in marine biosensing. Cas12a (Cpf1) recognizes a PAM sequence in double-stranded DNA, forms an R-loop, executes site-specific *cis*-cleavage, and then indiscriminately *trans*-cleaves quenched single-stranded DNA (FQ ssDNA) reporters. Cas13a binds its RNA target via crRNA without a PAM requirement, undergoes a HEPN-domain conformational change, and *trans*-cleaves single-stranded RNA reporters. Cas14 recognizes single-stranded DNA in a PAM-independent manner and, in an aptasensor configuration, couples target binding to a conformational reporter switch, extending CRISPR detection to non-nucleic-acid analytes.

Beyond direct nucleic acid detection, the integration of synthetic biology components has extended these systems to non-nucleic acid targets, substantially broadening the range of marine pollutants accessible to this technology [14]. Specifically, coupling CRISPR-Cas12a with aptamers, DNAzymes, and bacterial allosteric transcription factors (aTFs) allows the systems to successfully translate chemical stimuli from metal ions, small molecules, and proteins into readable nucleic acid signals [14]. For instance, aTFs can translate conformational changes induced by small molecules into a readable double-stranded DNA output that triggers Cas12a *trans*-cleavage, providing a highly adaptable framework for detecting diverse environmental targets [14,20]. The mechanistic differences among these three CRISPR-Cas effector systems are composed in Figure 2.

### 2.2. Microfluidic Platform Architectures

Microfluidics encompasses the precise manipulation of fluids within channels or structures at the sub-millimeter scale, enabling the miniaturization and integration of multiple analytical functions—including sample processing, separation, reaction, and detection—onto a single chip [12]. For ocean monitoring specifically, microfluidic technology confers five key advantages: high sensitivity enabling the detection of trace-level pollutants; high throughput allowing rapid analysis of large sample numbers; miniaturization resulting in portable and deployable devices suitable for both laboratory and field applications; low reagent consumption reducing cost and environmental impact; and the potential for automation simplifying complex analytical procedures [12]. Four main microfluidic architectures are relevant to CRISPR-integrated marine sensing, each with distinct operational characteristics. These four architectures were selected, out of the broader universe of microfluidic formats, because each represents a distinct fluid actuation and reaction-compartmentalization mechanism—continuous open-channel flow, discrete droplet compartmentalization, capillary-driven passive flow through porous media, and centrifugally driven (“lab-on-a-disc”) flow—that has been experimentally coupled with CRISPR-Cas *trans*-cleavage detection in the peer-reviewed literature identified through the search strategy in Section Literature Search Methodology, spanning the full range from laboratory-grade quantitative platforms (droplet-digital) to fully equipment-free field formats (paper/LFA). This classification is orthogonal to the specific CRISPR effector used, which is treated as an independent variable in Section 3. Other microfluidic formats, such as valve-based and acoustofluidic chips, exist but have not yet been integrated with CRISPR-Cas readout in the marine-monitoring literature identified by this review at the time of writing, and are therefore outside the scope of the comparison below.

Continuous-flow microfluidics features permanently open channels through which reagents flow in a controlled, sequential manner, making it well-suited to multi-step biochemical reactions such as nucleic acid extraction, isothermal amplification, and CRISPR reporter cleavage in a single linear workflow [11,22]. The most widely used substrate for continuous-flow devices is polydimethylsiloxane (PDMS), which offers optical transparency, gas permeability, and biocompatibility [23]; PDMS microfluidic chips have demonstrated significant application potential in marine and aquaculture environments, enabling high-throughput, high-efficiency, and high-precision detection of pathogenic microorganisms in aquatic systems [24]. Droplet-based microfluidics uses immiscible oil and aqueous phases to generate discrete picoliter-to-nanoliter droplets, each functioning as an isolated reaction chamber [25]. This architecture provides high measurement frequency and markedly reduced reagent consumption—demonstrated at 2.8 mL per day when measuring every ten seconds—enabling continuous, autonomous in situ monitoring with orders-of-magnitude greater reagent efficiency than conventional flow-through sensors [25]. When coupled with CRISPR *trans*-cleavage reporters, droplet compartmentalization additionally enables digital absolute quantification of target nucleic acids at the single-molecule level, a critical capability for detecting low-abundance ARGs and pathogenic eDNA in open ocean samples [11].

Paper-based microfluidic analytical devices (µPADs) exploit capillary action through porous cellulose matrices to drive fluid flow passively without pumps or external power [23], and since their first report in 2007 [26] have continued to gain attention as promising tools for point-of-care diagnostics due to their low cost, portability, ease of operation, and design flexibility [23]. Vertical-flow paper configurations, in which a stack of paper layers rather than a single plane handles sample transport, can manage higher sample loads, reduce detection artifacts arising from ionic fluctuations and organic load, and enable multiplex pollutant screening in a compact format—properties directly relevant to the high-salinity, particle-rich matrices of seawater samples [27]. Lateral flow assay (LFA) strips, a subset of paper-based microfluidics, enable naked-eye or smartphone-readable binary readout and have been extensively coupled with CRISPR *trans*-cleavage reporters to produce portable, equipment-free diagnostic strips [11]. Each architecture presents distinct trade-offs in sensitivity, throughput, quantification capability, and compatibility with marine matrices; these are systematically compared in the context of specific CRISPR integration strategies in Section 3.

### 2.3. Marine Detection Targets: Characteristics and Analytical Challenges

Four primary classes of marine detection targets dominate the scope of current environmental surveillance. Each presents distinct physicochemical and biological properties that impose specific analytical demands on any detection platform.

Heavy metals —mainly lead (Pb), cadmium (Cd), mercury (Hg), arsenic (As), copper (Cu), and zinc (Zn)—represent persistent inorganic pollutants that bioaccumulate through the marine food chain, posing serious threats to marine ecosystems and human health [28]. Detection of these ions in seawater presents compounded analytical challenges. Metal speciation—the distribution of a given element across free ionic, complexed, and particulate forms—varies dynamically with environmental factors such as temperature, pH, and the presence of organic ligands. Consequently, measuring the total metal concentration alone is insufficient to accurately assess true bioavailability and toxicity. Furthermore, detecting these metals at the ultra-low concentrations characteristic of oceanic waters (often <10^−8^ M) requires techniques capable of isolating the metal from the high ionic strength matrix of seawater, which otherwise causes competitive binding interference [29].

Harmful algal bloom (HAB)-derived biotoxins constitute the second target class, encompassing structurally diverse molecules produced by toxigenic phytoplankton. These include water-soluble toxins such as saxitoxin (STX) and domoic acid (DA), responsible for paralytic and amnesic shellfish poisoning respectively, as well as lipophilic biotoxins like okadaic acid (OA) and dinophysistoxins (DTXs), which are associated with diarrhetic shellfish poisoning [30]. The detection of these compounds is complicated by their extreme structural diversity and the existence of multiple toxic analogues within each family. The saxitoxin family alone comprises over 50 analogues with widely varying toxicity equivalence factors, demanding detection platforms capable of recognizing the full analogue spectrum rather than a single congener to accurately assess total toxicity [31]. Furthermore, the complexity of shellfish tissue and seawater matrices requires robust extraction and clean-up steps to prevent non-specific moieties from masking target analytes, severely complicating the development of rapid, point-of-site testing (POST) diagnostics for field use [31].

Pathogenic microorganisms—including *Vibrio* species (e.g., *V. harveyi*, *V. anguillarum*), aquaculture viruses (e.g., decapod iridescent virus 1 and white spot syndrome virus), and opportunistic bacteria like *Aeromonas* and *Pseudomonas*—form the third detection target class [32]. Traditional pathogen detection relies heavily on off-site diagnostic laboratories and invasive sampling, requiring the capture and handling of farmed animals. Analytically, the biological nature of these targets demands field-compatible tools that can non-invasively detect environmental DNA or RNA (eDNA/eRNA) directly shed into the water column, allowing for early intervention before mass mortality events occur [32]. A critical challenge in pathogen surveillance is the need for nucleic acid-level sequence specificity to discriminate between pathogenic and non-pathogenic strains of the same genus. This is a precision that traditional immunoassays cannot reliably achieve, but which is increasingly being solved by integrating CRISPR-Cas technologies with isothermal amplification (such as RPA or LAMP) [32].

Antibiotic Resistance Genes (ARGs) represent the fourth and analytically most complex detection target [33]. The aquatic resistome is highly dynamic; ARGs such as *catA2*, *tet(B)*, *blaTEM*, and *mecA* are disseminated across commensal and pathogenic bacteria via horizontal gene transfer (HGT) mechanisms including conjugation, transformation, and transduction [33]. This rapid environmental spread is heavily mediated by mobile genetic elements (MGEs), including transposons, integrons, and highly transferable conjugative plasmids like the multidrug-resistant pAQU-MAN [33]. Furthermore, ARGs evolve and persist not only in antibiotic-heavy aquaculture environments but also in pristine deep-sea ecosystems, serving as a vast natural reservoir of resistance mechanisms [34]. The multiplicity of resistance gene families and the compounding issue of “co-selection”—where heavy metals, microplastics, and agricultural pesticides promote ARG proliferation even without antibiotics—means that effective surveillance cannot rely on single-target assays [35]. Overcoming these challenges requires broad-spectrum, multiplexed nucleic acid strategies or advanced metagenomic and metatranscriptomic sequencing capable of comprehensively analyzing the entire microbial community and the evolutionary mobility of plasmids [32,34].

## 3. Microfluidic-CRISPR Integration Strategies

**Figure 3:**
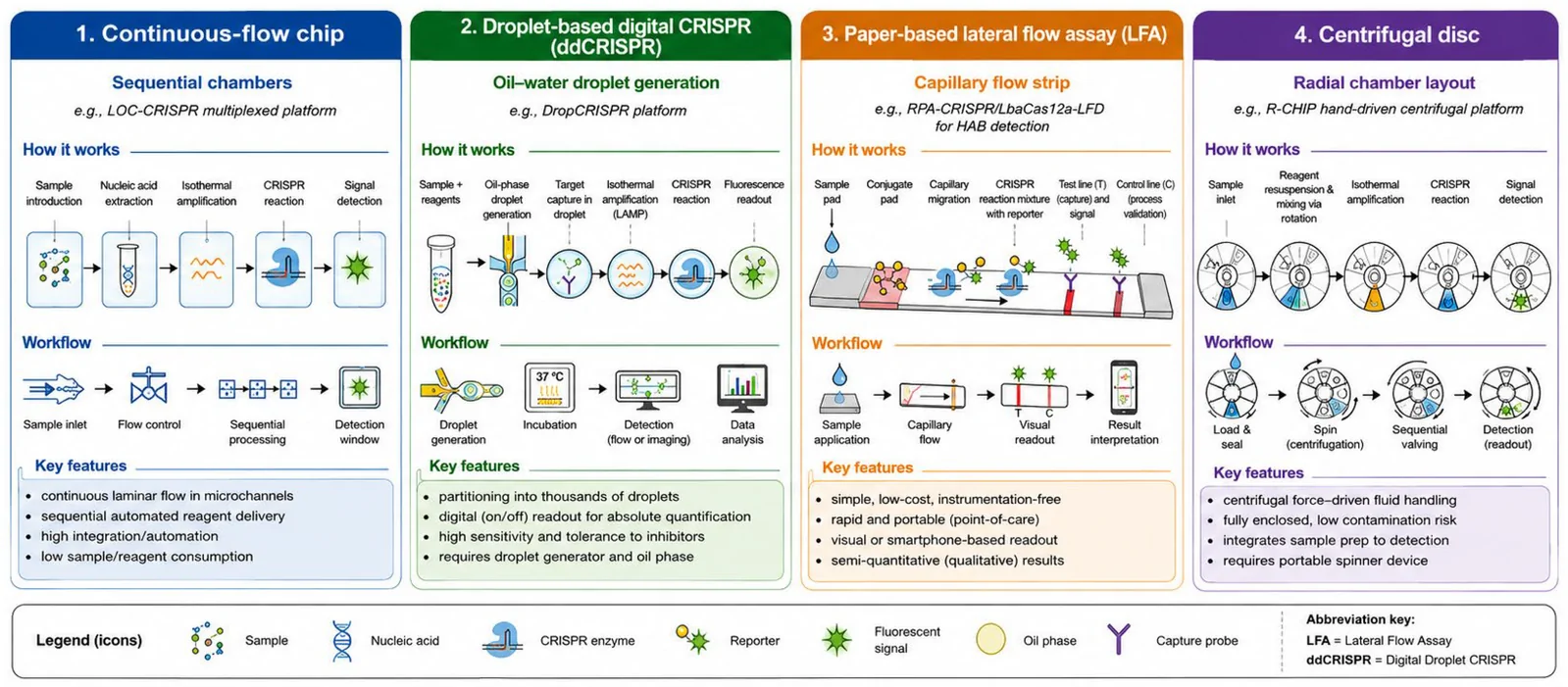
Comparison of the four principal microfluidic architectures used for CRISPR-integrated marine sensing, each annotated with a representative reported platform. (1) Continuous-flow chips perform sequential sample introduction, nucleic acid extraction, isothermal amplification, and CRISPR reaction in series along open microchannels, exemplified by the LOC-CRISPR multiplexed platform [36]. (2) Droplet-based digital CRISPR (ddCRISPR) partitions the reaction into thousands of picoliter oil-encapsulated droplets for digital, absolute quantification, exemplified by the DropCRISPR platform [37]. (3) Paper-based lateral flow assays (LFA) use capillary wicking through a test/control line strip for equipment-free, naked-eye or smartphone readout, exemplified by the RPA-CRISPR/LbaCas12a-LFD system developed for harmful algal bloom (HAB) detection [38] (4) Centrifugal (disc-based) platforms use rotationally driven, sequentially valved chambers to fully enclose and automate reagent handling, exemplified by the R-CHIP hand-driven centrifugal platform [39]. For each architecture, the operating principle, representative workflow, and key performance features are summarized; representative examples are illustrative of each architecture class and are not exhaustive.

The integration of CRISPR-Cas diagnostics with microfluidic platforms has produced four principal architectural strategies, whose respective trade-offs were introduced in Section 2.2. These strategies—continuous-flow chip integration, droplet-based digital CRISPR, paper-based and lateral flow CRISPR assays, and centrifugal microfluidic platforms—are reviewed here in the context of their specific suitability for field-deployable ocean sensing. These four architectures are compared in Figure 3.

### 3.1. Continuous-Flow Chip Integration

Continuous-flow microfluidic chips represent a comprehensive integration strategy for CRISPR-based biosensing, successfully automating nucleic acid extraction, isothermal amplification, and CRISPR-Cas detection within a single, fully enclosed device [36,40]. This integration directly overcomes the persistent barriers associated with traditional two-step CRISPR diagnostics, most notably the high risk of aerosol cross-contamination during amplicon transfer and the dependency on complex manual pipetting [36]. To facilitate automated, pump-free workflows suitable for point-of-care and field deployment, various fluid actuation mechanisms have been engineered. For instance, rotary valve and integrated plunger-assisted designs enable on-chip fluid routing, mixing, and magnetic bead-based nucleic acid washing by physically manipulating internal pressures and channel openings [41]. Alternatively, miniaturized devices utilize push-button air pouches to physically isolate the isothermal amplification and CRISPR detection steps; the amplified products are safely stored until manual compression generates the pressure difference needed to drive the fluid through a hydrophobic channel into the detection chamber [42]. For marine and aquaculture sensing, centrifugal and capillary forces offer a highly robust actuation method. The RPA-CRISPR/Cas12a-coupled centrifugal microfluidic biosensor (ORCMB) demonstrates this by relying on rapid centrifugation (1300 rpm) to mix test solutions with lyophilized reagents, followed by low-speed rotation (300 rpm) to trigger capillary action, smoothly drawing the fluid into terminal reaction chambers [43].

A central engineering challenge in continuous-flow chip design is managing the kinetic incompatibility between isothermal amplification and CRISPR *trans*-cleavage when both reactions operate simultaneously [40,43]. If Cas enzymes are activated too rapidly, their indiscriminate cleavage activity destroys the amplification templates and primers prematurely, which starves the amplification reaction and drastically reduces the final signal [40,43]. While spatial isolation using internal ledges or air pouches effectively bypasses this issue by separating the steps temporally [40,42], continuous-flow platforms must also prevent cross-contamination between parallel testing zones. In multiplexed arrays like the LOC-CRISPR platform, physical fluidic barriers utilizing low-viscosity dimethicone silicone oil are injected after the reaction mixtures are distributed, completely sealing the individual compartments to prevent free diffusion and ensure the specific identification of distinct viral targets or variants [36].

To achieve true “one-pot” integration without relying on physical barriers, continuous-flow chips for marine pathogen monitoring have successfully optimized biochemical parameters to modulate CRISPR cleavage kinetics [40,43]. In the centrifugal ORCMB platform designed for the detection of *Vibrio parahaemolyticus*, researchers intentionally utilized crRNAs targeting suboptimal PAM sites (such as CTTG) to artificially attenuate the Cas12a enzyme’s activation rate. This controlled kinetic delay creates a critical temporal window that allows the recombinase polymerase amplification (RPA) to exponentially amplify the marine pathogen’s target *toxR* gene before Cas12a cleavage can dominate and halt the reaction [43]. By pairing this biochemical tuning with a highly rigid polycarbonate (PC) microfluidic architecture featuring sacrificial chambers—which cleanly cut off excess fluid to lock the final reaction volume exactly at 6.25 μL—the chip guarantees highly consistent reaction conditions [43]. Furthermore, by preloading a gradient of standard plasmids directly onto parallel reaction chambers within the chip, the system can concurrently construct a standard calibration curve during the sample analysis run [43]. This on-chip, in-run calibration methodology successfully achieves a reliable quantitative linear range of 10^0^ to 10^4^ copies/μL (comparable to qPCR performance), demonstrating that continuous-flow CRISPR chips are a highly viable and robust solution for quantitative, field-deployable marine pathogen surveillance [43].

### 3.2. Droplet-Based Digital CRISPR (ddCRISPR)

Droplet-based digital CRISPR (ddCRISPR) represents a powerful diagnostic paradigm by integrating the high sequence specificity of CRISPR effectors with the absolute quantification capabilities of droplet digital microfluidics [37]. This platform functions by partitioning a bulk sample into thousands to millions of independent picoliter-scale microdroplets, effectively isolating single target molecules within discrete reaction units [37]. The statistical foundation of ddCRISPR is the Poisson distribution, which allows for the absolute determination of target concentrations based on the ratio of “positive” (fluorescent) to “negative” (dark) droplets, eliminating the need for external standard curves or calibration [44]. For marine environmental monitoring, this digital counting approach is particularly advantageous as it overcomes the quantitative biases and fluorescence quenching common in bulk assays when dealing with inhibitory substances or variable sample matrices [37].

Two primary strategies have been established for ddCRISPR: amplification-assisted and amplification-free [37]. Amplification-assisted platforms co-encapsulate isothermal methods such as recombinase polymerase amplification (RPA), loop-mediated isothermal amplification (LAMP), or rolling circle amplification (RCA) with CRISPR reagents to enhance the signal-to-noise ratio and enable clearer thresholding [37]. For instance, LAMP-assisted digital CRISPR—as demonstrated by the DropCRISPR platform—operates at near-ambient temperatures (60–65 °C for LAMP, followed by room-temperature CRISPR cleavage via picoinjection), which preserves droplet stability and facilitates field-deployable configurations [45]. Conversely, amplification-free ddCRISPR leverages the intrinsic sensitivity of Cas12 or Cas13 enzymes, where the target-triggered *trans*-cleavage of thousands of reporter molecules generates an ultralocalized fluorescence burst within the picoliter confinement [37]. These amplification-free methods simplify workflows, reduce reagent consumption, and mitigate the risk of cross-contamination, achieving single-molecule resolution for viral and bacterial biomarkers [46].

The application of ddCRISPR to environmental surveillance, particularly for antibiotic resistance gene (ARG) monitoring, has demonstrated superior sensitivity compared to conventional methods [47]. In complex wastewater matrices—which share similarities with particulate-rich marine environments—a CRISPR-enriched metagenomic approach was able to detect up to 1189 more ARGs than standard next-generation sequencing [47]. This methodology effectively lowered the detection limit from 10^−4^ to 10^−5^ relative abundance as quantified by qPCR [47]. Furthermore, systems like DropCRISPR have achieved detection limits as low as 10^2^ CFU/mL for live bacteria in complex, non-extracted samples like raw milk, highlighting a matrix-tolerant performance critical for high-salinity seawater applications [45].

To transition this technology toward autonomous ocean monitoring, significant progress has been made in “sample-to-answer” integration [48]. Advanced microfluidic chips now integrate IFAST-based (immiscible filtration assisted by surface tension) nucleic acid extraction with one-pot digital RPA/CRISPR, achieving quantification of targets like SARS-CoV-2 within 50 min at a sensitivity of 1 copy/μL [48]. These systems are increasingly paired with smartphone-based imaging and cloud-based deep learning algorithms to automate droplet segmentation and positive signal identification [48]. However, persistent challenges for marine field deployment include droplet instability under extreme temperature fluctuations and the impact of seawater salinity on surfactant performance, which can lead to droplet fusion or evaporation [37]. Future engineering must focus on enhancing device portability, automation, and the development of salt-tolerant microfluidic architectures to ensure robust performance in remote marine environments [37].

### 3.3. Paper-Based and Lateral Flow CRISPR Assays

Paper-based microfluidic platforms represent the most field-accessible integration architecture for CRISPR-based marine sensing, offering equipment-free, low-cost, and disposable operation suited to resource-limited coastal and offshore monitoring environments. The SHERLOCK platform, first introduced by Gootenberg et al., demonstrated the potential of Cas13a for RNA-based diagnostics; subsequent iterations integrated multiplexing by combining Cas13a, Cas12a, and Csm6, achieving a limit of detection of 2 aM while eliminating the need for sophisticated laboratory equipment and enabling lateral flow readout [49]. The complementary DETECTR platform harnessed Cas12a’s *trans*-cleavage activity for DNA detection, collectively establishing a family of paper-compatible CRISPR diagnostic architectures that underpin current marine biosensing development [50,51].

Three paper-based device formats have been applied to CRISPR marine detection: µPADs, lateral flow dipstick (LFD) assays, and integrated microfluidic-LFA hybrid chips. Multiplex LFAs represent one of the most significant advances in rapid diagnostics, combining multiple detection labels and amplification methods to achieve simultaneous multi-analyte detection within a single strip format suitable for both clinical and environmental monitoring [52]. The most directly marine-relevant demonstration of LFD-CRISPR integration is the RPA-CRISPR/LbaCas12a-LFD system developed for HAB detection: this method integrates RPA with CRISPR-LbaCas12a and lateral flow dipstick technology, enabling visual readout of results with a detection limit of 5 × 10^−6^ pg μL^−1^ for genomic DNA under laboratory conditions and 2.82 × 10^1^ cells mL^−1^ in simulated environmental seawater samples, with high specificity confirmed against closely related non-target algal species [38]. This was preceded by a landmark marine application: Wang et al. developed a CRISPR-Cas12a method for *Karenia mikimotoi* detection—a dinoflagellate responsible for highly damaging HAB events—using the internal transcribed spacer (ITS) region as target, with RPA preamplification and lateral flow dipstick readout achieving sensitive and specific detection within one hour, establishing proof-of-concept for field-deployable CRISPR-LFA HAB surveillance [53].

For pathogen detection, CRISPR-integrated LFA systems have been demonstrated for diverse aquatic pathogens: a Cas12a-based immunochromatographic strip achieved a limit of detection of 20 copies per reaction within one hour for African swine fever virus with full agreement with PCR gold standard across a field sample pool of 149 animals, illustrating the direct applicability of LFA-CRISPR to high-throughput aquaculture surveillance [54]. Note that ASFV is a livestock or veterinary pathogen rather than a marine target; it is included here solely as a methodological illustration of Cas12a-LFA workflow performance, not as marine validation evidence. The SHERLOCK/Cas13a-based CRISPR-LFA format has been applied to aquatic RNA virus detection—including grass carp reovirus (GCRV), a freshwater aquaculture pathogen included here as a further non-marine methodological example—through RPA-T7-Cas13a coupling, demonstrating that RNA-targeting capability extends CRISPR-LFA applicability beyond DNA-only targets to the full spectrum of marine viral pathogens [55].

Despite their field accessibility advantages, paper-based CRISPR platforms face three specific limitations in marine deployment. First, limitations remain regarding long-term reagent stability in lyophilised format and environmental durability under varying humidity and temperature conditions [49]—conditions that are particularly severe in ocean environments where humidity is consistently high and temperatures fluctuate with depth and season. Second, the binary yes/no readout of most LFA formats lacks the quantitative capability required for regulatory compliance monitoring, where specific concentration thresholds must be confirmed rather than merely exceeded. Third, the high salt and particulate load of seawater can disrupt nitrocellulose membrane flow kinetics, potentially generating false positives through non-specific probe binding—a challenge currently unaddressed in the marine-specific paper-CRISPR literature. Vertical-flow paper configurations and pre-integrated desalination membranes represent promising engineering approaches to mitigate these seawater-specific matrix effects.

### 3.4. Centrifugal Microfluidic Platforms

Centrifugal or disc-based microfluidic platforms use rotational force to drive sequential fluid operations through radially arranged chambers, enabling multi-step sample processing without external pumps or pressure sources. Core advantages of the centrifugal platform include the elimination of pneumatic interfaces and pumps, liquid handling independent of sample properties, full integration, automation, and miniaturisation—properties directly suited to field-deployable marine pathogen surveillance where infrastructure is unavailable [56]. The operational principle relies on pseudo-forces generated during disc rotation or oscillation to drive reagents through syphon valves into sequentially activated reaction chambers, enabling timed release of lysis buffers, amplification reagents, and CRISPR detection mixtures without manual pipetting.

The most directly environmentally relevant demonstration of centrifugal microfluidic capability is the centrifugal microfluidic disc (CD) developed for virus quantification in environmental water matrices: this portable system integrates sample concentration, purification, and droplet digital reverse transcription LAMP as a lab-on-a-chip system, with all assay steps completed in less than 1.5 h; on-disc sample preparation procedures were shown to be comparable to traditional in-tube methods, establishing proof of concept for autonomous waterborne pathogen detection [57]. For marine-specific pathogen targets, the RPA-CRISPR/Cas12a centrifugal biosensing system applied to *Vibrio parahaemolyticus* represents a direct translation of this architecture: using a portable centrifugal microfluidic nucleic acid testing device, one-pot RPA-CRISPR reaction conditions were systematically optimised using the *toxR* gene as target, achieving sensitive quantitative detection with performance comparable to qPCR and acceptable repeatability and stability under field-relevant conditions [43].

The most advanced centrifugal CRISPR integration to date is the R-CHIP system, which eliminates the need for any external instrumentation through hand-driven centrifugation: R-CHIP integrates RPA isothermal amplification, CRISPR/Cas12a detection, hand-driven microfluidics, and a ResNet-18 deep learning artificial intelligence platform into a single portable device; active and passive valves enable sample preprocessing and on-demand quantitative reagent release under low-speed manual operation, achieving detection limits of 10^−17^ M for HPV-16 and 10^−18^ M for HPV-18 with over 95% accuracy across 300 clinical samples in under one hour [39]. The deep learning image analysis component of R-CHIP is particularly relevant to marine deployment—automated fluorescence droplet segmentation and positive signal classification eliminates the need for trained operators, enabling community-based or autonomous buoy-mounted surveillance without laboratory personnel.

Despite these advances, centrifugal CRISPR platforms face specific challenges for marine field deployment. Current centrifugal microfluidic applications are mainly designed for laboratory-grade samples such as blood, lacking targeted solutions for the pretreatment of complex water samples, and rely on sophisticated temperature control equipment that constrains their field applicability [56]. For marine samples specifically, the high particulate and organic load of seawater can clog the filtration membranes and syphon valves integral to centrifugal disc operation, while the density differences introduced by salinity alter the centrifugal force calculations underlying fluid routing—engineering challenges that must be addressed before centrifugal CRISPR platforms can be reliably deployed for open-ocean monitoring. Integration of pre-disc desalination and filtration cartridges, combined with salt-tolerant RPA and Cas12a reaction buffer formulations, represents the most promising path toward marine-compatible centrifugal CRISPR diagnostics.

## 4. Marine Applications of Microfluidic-CRISPR Integrated Systems

### 4.1. Heavy Metal Detection

**Overview.** The detection of heavy metal ions—principally Pb^2+^, Cd^2+^, Hg^2+^, and As^3+^—in marine matrices presents a fundamental biochemical challenge for CRISPR-based systems: these analytes are non-nucleic acid targets, and CRISPR-Cas effectors have no intrinsic recognition capacity for inorganic ions. Three transduction strategies have been developed to bridge this gap, each converting a metal ion-binding event into a nucleic acid signal that activates Cas12a *trans*-cleavage: aptamer-based signal switching, DNAzyme-mediated catalytic release, and allosteric transcription factor (aTF) regulation.

**Representative platforms.** Aptamer-based CRISPR detection exploits the conformational change undergone by metal ion-specific aptamers upon target binding to generate or liberate a Cas12a-activating ssDNA trigger. In a representative SDA-CRISPR/Cas12a aptasensor for Cd^2+^, the aptamer initiates strand displacement amplification (SDA) in the absence of cadmium, generating abundant ssDNA that activates Cas12a and produces high fluorescence output; the presence of Cd^2+^ curtails SDA efficiency by sequestering the aptamer, resulting in a significant reduction in fluorescence—achieving selective detection at 60 pM with performance validated in real water samples [58,59]. For Hg^2+^, a structurally distinct approach exploits T–Hg^2+^–T base pairing: a two-segment poly-T DNA activator forms a hairpin structure via T–Hg^2+^–T coordination in response to mercury, suppressing Cas12a *trans*-cleavage activity; the system achieves a detection limit of 0.372 nM with high Hg^2+^ selectivity, with *trans*-cleavage efficiency decreasing 3.03-fold as Hg^2+^ concentration increases to 40 nM, and has been validated in river water, tap water, and seawater samples [60].

DNAzyme-CRISPR cascade systems harness the metal ion-dependent catalytic cleavage activity of DNAzymes to produce ssDNA activators for downstream Cas12a detection. By coupling the GR-5 DNAzyme—which cleaves its substrate in the presence of Pb^2+^—with CRISPR/Cas12a as a signal enhancer, the released ssDNA from DNAzyme cleavage directly activates Cas12a *trans*-cleavage; after optimisation, this DNAzyme-CRISPR achieves a colorimetric detection limit of 0.47 nM and an electrochemical detection limit of 0.14 pM for Pb^2+^, representing a fivefold improvement in sensitivity compared to DNAzyme-only detection [61]. The functional alliance of DNAzymes with CRISPR/Cas12a offers two compounding advantages: DNAzymes function as allosteric modules to precisely translate metal ion recognition into nucleic acid signals, while the intrinsic nuclease activities of both systems contribute synergistically to cascade signal amplification, substantially improving detection sensitivity beyond either component alone [62]. A triple amplification strategy integrating SDA cycling with GR-5 DNAzyme and CRISPR/Cas12a electrochemical detection achieved an impressive LOD of 0.02 pM for Pb^2+^—among the lowest reported for any Pb^2+^ biosensor [63].

Microfluidic integration has advanced both the throughput and field applicability of heavy metal detection, with lab-on-a-chip platforms providing a primary pathway for the development of portable CRISPR/Cas12a biosensors. A representative centrifugal microfluidic system designed for the automated detection of multiple heavy metal ions utilized an aptamer-based gold nanoparticle (AuNP) colorimetric assay—rather than a CRISPR-based mechanism—to demonstrate the simultaneous quantification of Pb^2+^, Hg^2+^, and As^3+^. This platform integrates automated reagent metering, sampling, and mixing within a single PMMA chip through the use of capillary valves and controlled rotational speeds. The system is capable of performing multi-ion analysis in under 30 min, achieving detection limits of 6.16 ppb (Pb^2+^; ≈29.7 nM), 4.97 ppb (Hg^2+^; ≈24.8 nM), and 5.24 ppb (As^3+^; ≈69.9 nM). While such microfluidic advancements improve on-site potential, the lack of validated seawater-specific protocols remains a critical challenge for direct marine deployment [64]. For seawater-specific deployment, a microfluidic system utilizing a GR/CeO_2_/Nafion composite membrane on a gold planar disc electrode was developed for the measurement of zinc ions (Zn^2+^) in marine environments. While the Nafion coating provides critical anti-interference capabilities against common seawater ions like Na^+^ and Cl^−^, the protocol does not enable direct seawater injection without dilution. Instead, for real-sample analysis, filtered seawater must be combined with an acetate buffer (HAc–NaAc) at a 1:1 ratio to create the optimal electrochemical environment for detection. This integrated platform features a curved hybrid mixing channel and utilizes square-wave voltammetry (SWV) to achieve a detection limit of 0.87 µg/L (Zn^2+^; ≈13.3 nM), which is well below the standards for drinking water and fishery quality [65]—a matrix-handling strategy directly applicable to CRISPR-integrated heavy metal microfluidic chips. A comprehensive review of microfluidic devices for heavy metal ion detection confirms that electrochemical and optical LOC platforms can achieve sub-ppb detection thresholds for Pb, Hg, As, Cd, and Cr, with the key remaining challenges being selectivity in complex multi-ion matrices and the absence of validated seawater-specific protocols [66].

Collectively, these studies demonstrate that heavy metal CRISPR biosensing has matured around three reproducible transduction chemistries, but with performance benchmarked almost exclusively against clean or spiked buffers rather than authentic seawater—underscoring the persistent gap between laboratory sensitivity and marine field readiness that motivates the comparison below.

**Figure 4:**
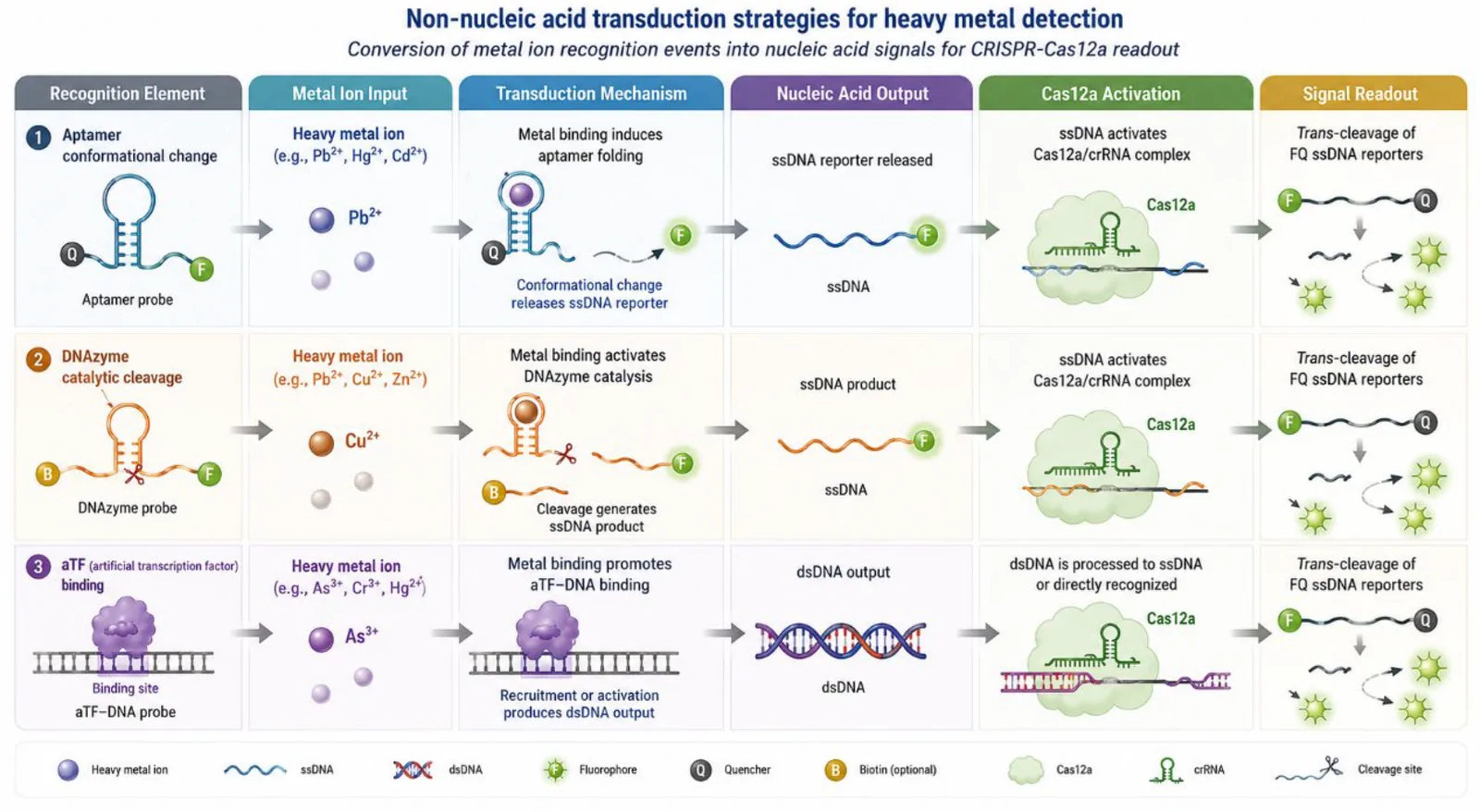
Non-nucleic-acid transduction strategies coupling heavy metal ion recognition to CRISPR-Cas12a readout. (1) Aptamer-based transduction: metal ion binding induces aptamer conformational change and release of a fluorophore-quencher (FQ) ssDNA reporter. (2) DNAzyme-based transduction: metal ion binding activates DNAzyme catalytic cleavage, generating an ssDNA product. (3) Allosteric transcription factor (aTF)-based transduction: metal ion binding promotes aTF-DNA binding, generating a double-stranded DNA (dsDNA) output. In all three strategies, the resulting nucleic acid output activates Cas12a *trans*-cleavage of FQ ssDNA reporters for signal generation. Because all three strategies converge on the same CRISPR readout module, future sensitivity gains are more likely to come from improving the upstream recognition element than from the CRISPR chemistry itself. The color coding distinguishes the three transduction strategies (blue, orange, and purple) and the common Cas12a activation and signal-readout modules (green and yellow, respectively).

**Critical comparison and marine engineering challenges.** Despite significant progress, two gaps remain specifically for marine CRISPR heavy metal detection. First, virtually all reported aptamer-CRISPR and DNAzyme-CRISPR systems have been validated in freshwater, tap water, or spiked laboratory buffers—not authentic seawater. The high Na^+^, Mg^2+^, and Ca^2+^ concentrations in seawater are known to disrupt G-quadruplex aptamer folding and DNAzyme catalytic rates, yet no systematic study has characterised this salinity-induced interference for CRISPR-coupled metal ion detection. Seawater validation is not intrinsically infeasible for this sensor class, however: a dual-colour whole-cell bacterial biosensor combining CadR- and MerR-regulated fluorescent reporters achieved direct detection of Cd(II), Pb(II), and Hg(II) in authentic seawater with only minor Hg(II)-specific salt interference [67]—demonstrating that seawater-compatible biological metal-ion recognition is achievable, even though no CRISPR-coupled platform has yet replicated this validation. Comparing the reported CRISPR platforms directly, DNAzyme-cascade and triple-amplification electrochemical designs achieve detection limits of 0.02–0.14 pM for Pb^2+^, outperforming aptamer-based fluorescence switching (60 pM for Cd^2+^) by two to three orders of magnitude—a gain attributable to catalytic DNAzyme turnover versus the single-binding-event stoichiometry that caps aptamer-based signal generation. This sensitivity advantage, however, comes at the cost of more complex electrode fabrication and calibration relative to the simpler tube-based fluorescence formats. Second, multiplexed simultaneous detection of two or more heavy metals in a single microfluidic-CRISPR assay has not been demonstrated for marine matrices, despite the co-occurrence of Pb^2+^, Cd^2+^, and Hg^2+^ as simultaneous threats in industrialised coastal zones. As illustrated in Figure 4, all three transduction strategies converge on the same underlying design logic: a metal-recognition event is converted into a nucleic acid trigger that Cas12a then amplifies into a fluorescent signal, meaning improvements to any one recognition element (aptamer, DNAzyme, or aTF) can in principle be paired with the same downstream CRISPR amplification module.

**Future perspective.** Closing the marine validation gap will likely require standardised seawater-matrix reference panels and a shift toward catalytic (DNAzyme- or enzyme-amplified) recognition chemistries capable of retaining sensitivity under high-ionic-strength conditions.

### 4.2. HAB-Derived Biotoxin Detection

**Overview.** HAB-derived biotoxins present a detection challenge qualitatively different from heavy metals: they are structurally complex organic molecules requiring both molecular recognition of specific toxin classes and, ideally, discrimination between multiple toxic analogues within the same family. CRISPR-based detection of these non-nucleic acid targets follows two parallel strategies—aptamer-coupled Cas12a systems for direct toxin quantification, and eDNA/eRNA-targeted CRISPR assays for early-warning bloom detection before toxin release.

**Representative platforms.** Direct toxin detection via CRISPR-aptasensor coupling has been demonstrated for microcystin-LR (MC-LR), the most prevalent and regulated cyanobacterial hepatotoxin. The MC-LR-Casor platform integrates MC-LR-specific aptamers conjugated to magnetic beads with blocker DNA, which is released upon MC-LR binding and activates a Cas12a-crRNA complex through its programmable *trans*-cleavage activity; signal readout is achievable via fluorometer or lateral flow strip, with output positively correlated to MC-LR concentration, achieving ultrasensitive parts-per-trillion limits of detection for on-site environmental monitoring [68]. For paralytic shellfish toxins, saxitoxin (STX)—the most acutely toxic PSP analogue—has been detected using various aptamer-based configurations (including CRISPR-Cas9 integrations) achieving LODs well below the WHO regulatory threshold of 80 μg STX equivalent per 100 g shellfish tissue [69]. Shellfish toxins span five poisoning categories—DSP, PSP, NSP, ASP, and CFP—encompassing analytes including okadaic acid, dinophysistoxins, saxitoxin and its alogues, domoic acid, brevetoxin, and ciguatoxin; routine monitoring must therefore address a structurally and chemically heterogeneous target landscape [69]. A LOAD (Lab-On-A-Disc) centrifugal microfluidic immunofluorescence platform demonstrated simultaneous detection of microcystin-LR, domoic acid, and saxitoxin within 30 min with less than 5 min of end-user interaction, combining recombinant antibody technology with centrifugally driven microfluidic liquid handling and an LED-photodiode optical sensing system for quantified fluorescence readout—establishing the proof-of-concept for multiplexed marine biotoxin detection on portable disc platforms [70]. While this platform used immunological rather than CRISPR recognition, its architecture is directly compatible with aptamer-CRISPR reporter substitution for enhanced programmability.

eDNA-targeted CRISPR assays represent the most marine-relevant HAB detection strategy, enabling early warning surveillance of bloom-forming species before toxin accumulation reaches harmful levels. Wang et al. developed the first CRISPR-Cas12a method for *Karenia mikimotoi* detection, targeting the internal transcribed spacer (ITS) region with RPA preamplification and LbCas12a-guided *trans*-cleavage, enabling both fluorescence and lateral flow dipstick readout and achieving species-specific detection within one hour—establishing the foundational framework for field-deployable CRISPR-LFA HAB surveillance [53]. This was extended to *Chrysotila dentata*, a haptophyte responsible for coastal aquaculture damage, using the RPA-CRISPR/LbaCas12a-LFD system validated in simulated seawater at 2.82 × 10^1^ cells mL^−1^ with confirmed specificity against closely related non-target algal species [38]. For toxigenic cyanobacteria, a portable RPA-CRISPR/Cas12a biosensing platform targeting the microcystin synthetase E (*mcyE*) gene enables early genetic detection of toxin-producing cyanobacteria before or during the early stages of bloom formation—providing critical predictive value that direct toxin analysis cannot, since MC production is cell-density dependent and typically triggered during peak cell proliferation [71]. Furthermore, the future integration of CRISPR-Cas systems with microfluidics has the potential to enable fast, high-throughput, and multiplexed detection across diverse HAB genera including *Alexandrium*, *Pseudo-nitzschia*, *Heterosigma*, *Microcystis*, and *Raphidiopsis*—a development that would substantially advance comprehensive marine bloom surveillance [72].

Collectively, these studies demonstrate two complementary but still-immature CRISPR strategies for HAB surveillance—direct toxin sensing and eDNA-based early warning—neither of which has been tested against a live bloom in authentic seawater, reinforcing the gap between laboratory-validated sensitivity and field-ready marine surveillance.

**Figure 5:**
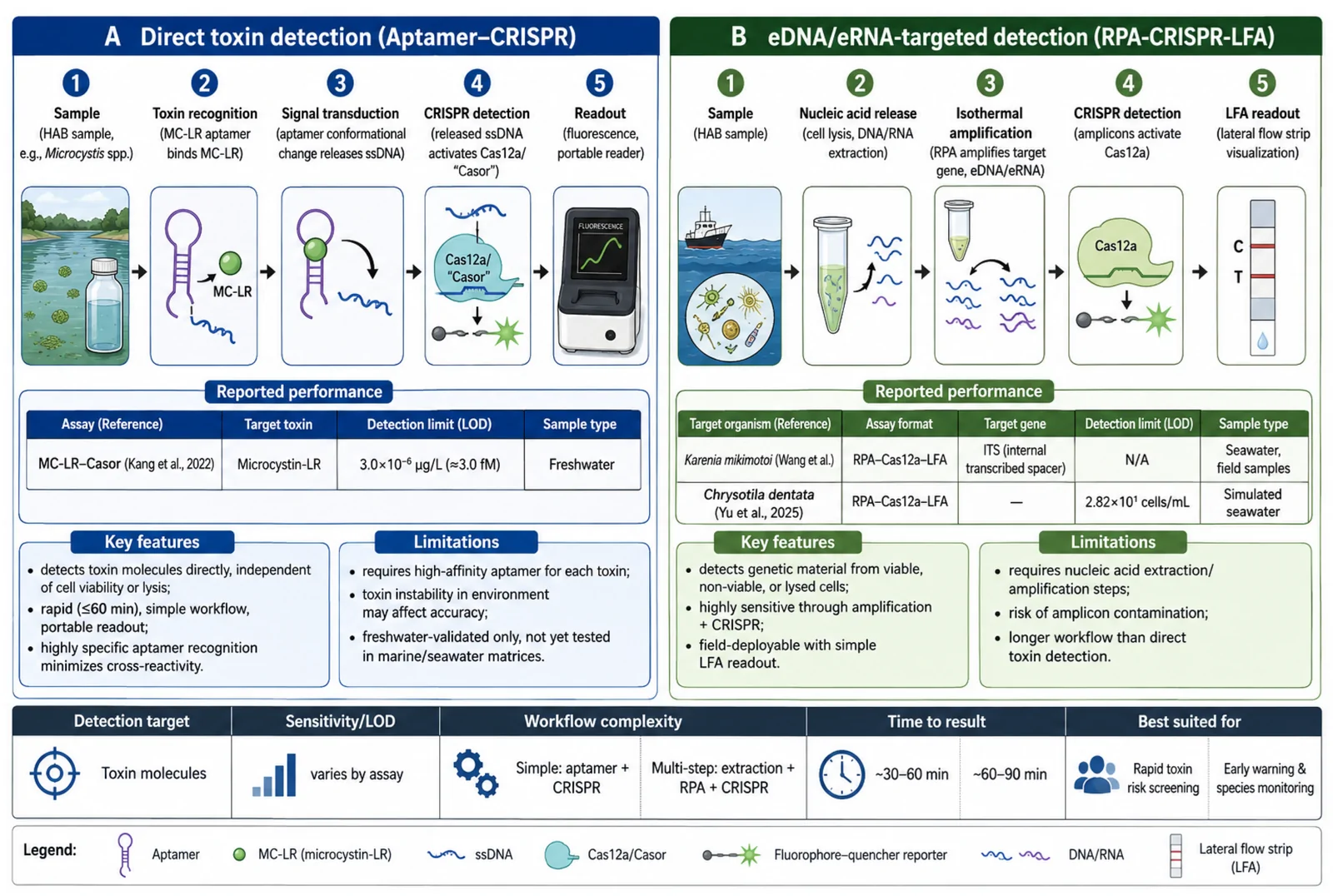
Comparison of two CRISPR-based strategies for harmful algal bloom (HAB) detection. (**A**) Direct toxin detection via an aptamer-CRISPR cascade, exemplified by the MC-LR–Casor assay [68], in which toxin-induced aptamer conformational change releases an ssDNA trigger for Cas12a *trans*-cleavage and fluorescence readout. (**B**) Environmental DNA/RNA (eDNA/eRNA)-targeted detection via RPA-CRISPR-lateral flow assay (LFA), exemplified by assays for *Karenia mikimotoi* (Wang et al.) [53] and *Chrysotila dentata* (Yu et al., 2025) [38], in which isothermal amplification of algal genetic material precedes Cas12a-mediated lateral flow readout. Representative reported detection limits, target genes, and sample types are summarized for each strategy alongside their relative advantages and limitations for field deployment. No platform yet combines both strategies, meaning current HAB biosensors must choose between assay speed and biological early-warning value rather than delivering both.

**Critical comparison and marine engineering challenges.** Despite this progress, three gaps remain for marine CRISPR HAB detection. First, no CRISPR-based platform has yet demonstrated simultaneous multiplexed detection of both the causative organism (eDNA target) and its associated toxin (small molecule target) in a single integrated assay—a capability that would transform HAB response from species monitoring to toxin risk assessment in one step. Second, the structural diversity of the saxitoxin family (>50 analogues) means that aptamer-based CRISPR detection of STX cannot reliably reflect total toxicity without multiplexing distinct analogue-specific aptamers (each linked to unique crRNA reporters), which has not yet been demonstrated. Third, all current CRISPR HAB biosensors have been validated in laboratory or simulated seawater conditions—none have been deployed during an actual bloom event for real-time field validation. As illustrated in Figure 5, the two strategies embody a fundamental trade-off between assay directness and biological relevance: aptamer-based toxin detection is faster and gives a direct exposure readout, whereas eDNA-based detection targets the causative organism and can therefore provide earlier warning, before toxin accumulation reaches hazardous levels.

**Future perspective.** A single integrated platform combining eDNA-based bloom-species screening with aptamer-based toxin quantification—and validated through an actual field bloom event—would represent the clearest path from laboratory demonstration to operational HAB early-warning systems.

### 4.3. Pathogenic Microorganism Detection

**Overview.** Marine pathogen detection represents the application area where CRISPR-Cas diagnostics have achieved the greatest translational maturity, with multiple field-validated systems demonstrated for aquaculture-relevant targets. The biological nature of these targets—nucleic acids shed directly into the water column as eDNA or eRNA—means that CRISPR effectors can engage them directly without the transduction intermediaries required for heavy metal or biotoxin detection, enabling the full sensitivity advantage of programmable *trans*-cleavage to be realised.

**Representative platforms.** Bacterial pathogen detection has been most extensively demonstrated for *Vibrio* species, which collectively represent the leading cause of aquaculture losses globally. Xiao et al. developed an RAA-CRISPR/Cas12a method for *Vibrio vulnificus*—a zoonotic aquatic pathogen with a human foodborne fatality rate of up to 50%—achieving a detection limit of two copies of genomic DNA per reaction within 40 min without sophisticated instrumentation, with confirmed specificity against non-target bacteria and high accuracy in spiked shrimp samples [73]. For *Vibrio parahaemolyticus*, the RPA-CRISPR/Cas12a centrifugal microfluidic biosensor targeting the *toxR* gene achieved quantitative detection at 6.08 copies/μL comparable to qPCR performance [43]. Application examples for microfluidics-based pathogen detection in aquaculture encompass a broad range of organisms beyond *Vibrio* spp., including *Aeromonas hydrophila*, *Edwardsiella tarda*, and *Pseudomonas aeruginosa*, establishing an early precedent for chip-based bacterial surveillance across a broad range of marine bacterial pathogens [32].

Aquaculture virus detection has been addressed across multiple economically critical targets. White spot syndrome virus (WSSV)—listed as a notifiable pathogen by the World Organisation for Animal Health and capable of causing up to 100% shrimp mortality within seven days—has been targeted by multiple CRISPR platforms. An RPA-CRISPR/Cas12a one-pot method for WSSV achieved a sensitivity of 10^1^ copies per reaction, overcoming the carry-over contamination risks of the earlier two-step DETECTR procedure by integrating RPA amplification and Cas12a cleavage into a single reaction tube with lateral flow strip readout [74]. The most advanced WSSV CRISPR platform combined isothermal amplification with fully field-deployable extraction: by pairing CRISPR detection with paper matrix-based nucleic acid extraction and lateral flow reporting, single-copy detection was achieved without sophisticated equipment or electricity—creating a truly point-of-care diagnostic applicable to remote aquaculture sites [75]. For RNA viruses, grass carp reovirus type 1 (GCRV) was detected using RPA-CRISPR/Cas13a targeting the *vp7* gene: this system achieved a detection limit of 7.2 × 10^1^ copies/μL with results readable via fluorescence signal, UV excitation visual fluorescence, and lateral flow test strip—providing three independent readout modalities suited to different field resource conditions [55].

Multiplexed pathogen detection represents an emerging frontier of CRISPR aquaculture diagnostics. A multiplex CRISPR-Cas assay exploited the orthogonal reporter specificity of Cas12a (ssDNA-linked ROX-quencher reporter) and Cas13a (ssRNA-linked FAM-quencher reporter) for simultaneous detection of WSSV and *Enterocytozoon hepatopenaei* (EHP) in penaeid shrimp, generating red, green, and yellow fluorescence for single-pathogen and co-infected samples respectively—demonstrating that dual-effector multiplexing with spectrally distinct reporters enables simultaneous multi-pathogen surveillance in a single reaction [76]. Recent comprehensive reviews confirm that CRISPR-Cas12a and Cas13a platforms now represent the most promising rapid detection technologies for aquatic microorganisms, with integrated comparison across sensitivity, specificity, speed, and field-deployability demonstrating advantages in speed, portability, and operational simplicity compared with conventional PCR and immunoassay workflows for aquaculture surveillance [77]. Comparing readout formats directly, paper-based LFA assays such as the WSSV platform (LOD of 1 copy) offer naked-eye interpretation well suited to field use but yield only qualitative results, whereas fluorescence-based tube assays for *Vibrio vulnificus* and GCRV retain quantitative resolution at the cost of requiring a benchtop reader. Dual-effector multiplexing (WSSV + EHP) only partially resolves this trade-off, since distinguishing red/green/yellow fluorescence channels still requires either a plate reader or, as demonstrated for authentic ocean water samples, a fully self-contained 3D-printed field platform.

Collectively, these studies demonstrate that pathogen detection is the most translationally mature of the four target classes, with several platforms already validated in real seawater or aquaculture samples—yet even here, quantitative readout and multi-pathogen scaling remain unresolved.

**Critical comparison and marine engineering challenges.** Despite this progress, three gaps remain for marine CRISPR pathogen detection. First, current CRISPR-based dual-readout systems are inherently restricted to qualitative analysis and cannot serve as a reliable quantitative biomarker for estimating initial nucleic acid concentrations. Second, sample preparation remains a critical bottleneck because complex environmental matrices contain amplification inhibitors, meaning assays still heavily rely on field-compatible nucleic acid extraction protocols. Third, scaling these assays to multi-pathogen panels remains a challenge because it requires orthogonal Cas effectors or microfluidic partitioning to control cross-talk between simultaneous reaction. To directly overcome these limitations, the integration of CRISPR-Cas systems with microfluidic Lab-on-a-Chip (LOC) platforms has emerged as an enabling solution. Microfluidic architectures not only enable automated on-chip sample preparation and spatial partitioning for extensive multiplexing but also facilitate precise kinetic control for accurate on-site quantification.

**Future perspective.** Pairing field-compatible extraction chemistry with orthogonal multi-effector multiplexing is the most direct route toward a quantitative, panel-based CRISPR pathogen sensor deployable directly from open ocean water.

### 4.4. Antibiotic Resistance Gene (ARG) Detection

**Overview.** ARG detection is arguably the most analytically demanding application within scope of this review, combining the nucleic acid targeting strengths of CRISPR-Cas with the formidable challenge of detecting low-abundance, structurally divers resistance gene families across complex saline matrices. Marine ARG surveillance matters beyond the coastal environment itself: horizontally mobile resistance genes that accumulate in marine sediments and biofilms can re-enter human-associated microbial communities through aquaculture products, recreational water contact, and the wider coastal food web, making the ocean both a reservoir and a transmission route for antimicrobial resistance in a One Health sense. Unlike the other three target classes, ARGs exist as mobile genetic elements distributed across phylogenetically diverse bacterial communities—meaning their detection cannot rely on a single conserved genomic locus but requires programmable, multiplexable nucleic acid recognition strategies capable of simultaneously targeting multiple resistance gene families. It is important to note, however, that this analytical demand has to date been characterised almost entirely using non-marine matrices (see below); direct evidence of CRISPR-ARG performance in authentic seawater remains absent.

**Representative platforms.** Single-target CRISPR-ARG detection has been validated in wastewater and freshwater matrices across several clinically critical resistance genes. A Cas12a-based one-step method leveraging RPA-coupled *trans*-cleavage detected four ARG markers—*sul1*, *qnrA-1*, *mcr-1*, and class 1 integrons (*intl1*)—in tap water, farm wastewater, and hospital wastewater with sensitivity and specificity matching qPCR, without complex equipment, laying an important basis for future ARG surveillance systems in the urban water cycle [78]. For portable field deployment, a LAMP-CRISPR/Cas12a biosensor for the *ermB* macrolide resistance gene achieved an LOD of 2.75 × 10^3^ copies/μL utilizing visual fluorescence or lateral flow strips, with results obtainable within 2 h [79]. A versatile and portable Cas12a platform detected resistance markers including *bla*CTX-M-15 and *floR* at less than 100 attoMolar (aM) of pure DNA fragments and less than 10^2^ CFU/mL in microbiological assays, with a total analysis time of 100 min and showing high concordance when compared to antibiotic susceptibility tests, PCR, and whole genome sequencing across clinical isolates [80].

Compared with the single-target assays above—which are simpler to deploy but detect at most one resistance gene family per reaction—multiplexed platforms trade portability for analytical breadth. Multiplexed CRISPR-ARG detection has been most powerfully demonstrated by the bCARMEN platform: bCARMEN combines modular CRISPR-Cas13-based nucleic acid detection with a droplet microfluidic system enabling thousands of simultaneous spatially multiplexed detection reactions at nanoliter volumes; the system detects and discriminates 52 clinically relevant bacterial species and key antibiotic resistance genes including *mecA*/*mecC*, *van* genes, *bla*KPC, *bla*NDM-1, *bla*VIM, *bla*IMP, *oxa48*-like, *bla*CTX-M-15, and *mcr1*, with 100% accuracy [81]. A simplified CARMEN v2 addressed field deployment barriers by using pre-loaded lyophilised microarrays with smartphone-based fluorescence readout, completing multiplexed ARG identification in under three hours—demonstrating a scalable architecture directly relevant to shipboard or coastal monitoring station deployment [81]. For marine-specific ARG surveillance, a CRISPR-Cas9-enriched next-generation sequencing approach detected up to 1189 more ARGs than standard metagenomic sequencing in complex wastewater matrices, with false negative and false positive rates of 2/1208 and 1/1208 respectively—establishing the principle that CRISPR-based enrichment dramatically increases ARG detection sensitivity in the complex matrix conditions analogous to marine water samples [47].

Collectively, these studies demonstrate that ARG detection is the most analytically demanding of the four target classes, with single-target assays trading marine relevance for simplicity and multiplexed platforms such as bCARMEN trading portability for analytical breadth—neither yet combining seawater validation with field-deployable multiplexing.

**Critical comparison and marine engineering challenges.** Despite these advances, five specific gaps limit current CRISPR-ARG systems for marine deployment. First, no CRISPR-ARG detection platform has been validated in authentic seawater—all reported systems use freshwater, wastewater, or clinical matrices. The high salinity and ionic competition of seawater are expected to inhibit RPA polymerase activity and disrupt Cas12a PAM recognition efficiency, yet these effects remain uncharacterised for ARG-targeting CRISPR assays. Second, the co-selection mechanisms identified in Section 2.3—whereby heavy metals and microplastics co-select for ARG-carrying bacteria—mean that effective marine ARG surveillance requires simultaneous multi-analyte platforms covering both chemical stressors and resistance gene markers, a cross-class multiplexing capability that no current microfluidic-CRISPR system has demonstrated. Third, the bCARMEN platform, while capable of detecting 14 key resistance determinants simultaneously with 100% clinical accuracy, requires DropArray chip infrastructure that is incompatible with autonomous marine sensor deployment [81]. Translating its multiplexing architecture into a compact, salt-tolerant, self-powered format represents the primary engineering challenge for ocean-scale ARG surveillance. Fourth, the marine resistome extends beyond free-living, planktonic ARG-carrying bacteria: horizontal gene transfer via conjugative plasmids, transposons, and integrons is substantially enhanced within surface-associated biofilms—including those that colonise net pens, fouling-release coatings, and unprotected microfluidic inlet channels—where high cell density and close physical contact promote conjugation frequencies well above those in the free water column [30,32]. This biofilm-associated ARG enrichment pathway has not been addressed by any CRISPR-based marine detection platform reviewed here, and its interaction with the biofouling challenges discussed in Section 7.2 represents a compounding, unaddressed risk for autonomous ARG biosensor nodes; the specific enrichment magnitudes reported for aquaculture-associated biofilms should be verified against the primary literature before quantitative claims are made in a final submission. Fifth, marine ARG monitoring lacks standardised regulatory thresholds—unlike heavy metals and biotoxins, no WHO or EU regulatory limits exist for ARG concentrations in coastal waters, limiting the translation of sensitive detection platforms into actionable management decisions.

**Future perspective.** Coupling cross-class multiplexing (ARGs alongside their heavy-metal and microplastic co-selectors) with biofouling-resistant sensor housings represents the key engineering step toward continuous, autonomous marine resistome surveillance.

### 4.5. Cross-Class Comparison of Marine CRISPR Biosensor Target Categories

The four target classes reviewed above differ substantially in recognition complexity, CRISPR platform maturity, marine-matrix validation, multiplexing capability, and readiness for field deployment. These differences highlight that analytical sensitivity alone is insufficient for assessing the practical suitability of a CRISPR-microfluidic biosensor for marine monitoring. Table 1 summarizes the key trade-offs across these five criteria.

## 5. Signal Transduction and Readout Strategies

The analytical performance of microfluidic-CRISPR integrated systems for marine monitoring is determined not only by the specificity and sensitivity of the CRISPR recognition event but critically by the efficiency and field-compatibility of the downstream signal transduction modality. Five principal readout strategies have been applied to CRISPR-based marine biosensing: fluorescence, electrochemical, colorimetric/lateral flow, surface-enhanced Raman scattering (SERS), and smartphone-integrated digital readout. Each presents distinct trade-offs in sensitivity, quantification capability, equipment requirements, and compatibility with saline marine matrices.

### 5.1. Fluorescence Readout

Fluorescence-based detection is the most widely used readout modality in CRISPR biosensing, exploiting the collateral *trans*-cleavage of fluorophore-quencher (FQ) reporter probes to generate a quantifiable signal upon target recognition. Two commonly used CRISPR-Cas detection modes exist: binding activity-based systems (Cas9 and dCas9) and cleavage activity-based systems (Cas12a, Cas12b, Cas13, and Cas14) [82]. For cleavage-based effectors—Cas12a, Cas13, and Cas14—fluorescence is generated through *trans*-cleavage of FQ ssDNA or ssRNA reporters, producing a signal directly proportional to target concentration [82]. In contrast, binding-based effectors such as Cas9 and dCas9 lack *trans*-cleavage activity and instead generate fluorescent signals through alternative mechanisms—including metal–organic framework (MOF)-mediated probe release upon strand displacement, or intercalating dyes such as SYBR Green I that fluoresce upon incorporation into double-stranded DNA [82]. For marine biosensing applications, the cleavage-based fluorescence modality is directly compatible with the portable fluorimeters and UV transilluminators demonstrated in aquaculture pathogen and ARG detection platforms reviewed in Section 4.3 and Section 4.4. However, future development should focus on visualising fluorescence signals to enable more convenient and user-friendly point-of-care testing, since current fluorescence detection systems still require stable excitation light sources and optical filters incompatible with fully autonomous ocean sensor configurations [83].

### 5.2. Electrochemical Readout

Electrochemical CRISPR biosensors transduce Cas12a *trans*-cleavage events into measurable changes in current, voltage, or impedance, offering quantitative readout without optical components. Four principal EC-CRISPR platforms—Cas9, Cas12a, Cas13a, and Cas14a—have been developed, each with distinct structural characteristics, biosensing mechanisms, and signal amplification strategies for both nucleic acid and non-nucleic acid target detection [84]. The E-CRISPR architecture exploits the *trans*-cleavage of surface-tethered hairpin DNA reporters labelled with electrochemical tags such as methylene blue, generating a quantifiable current reduction upon target recognition—enabling integration onto miniaturised electrode configurations compatible with portable potentiostats. For marine-specific application, an electrochemical CRISPR/Cas12a biosensor for *Vibrio parahaemolyticus* coupled PCR amplification with E-CRISPR using a hairpin DNA probe immobilised on a gold electrode; upon target recognition, Cas12a *trans*-cleavage cleaves the probe and detaches the methylene blue tag from the electrode surface, generating a quantifiable reduction in current for sensitive and selective detection, with the platform demonstrating strong miniaturisation potential and compatibility with real-time field monitoring [85]. The key advantage of electrochemical readout for marine deployment is its independence from optical excitation—miniaturised potentiostats can operate on battery power and are unaffected by the turbidity and chromophoric dissolved organic matter that compromise fluorescence and colorimetric readout in natural seawater.

### 5.3. Colorimetric and Lateral Flow Readout

Colorimetric and lateral flow assay (LFA) readout modalities offer the most accessible field-deployable detection format for CRISPR-based marine sensing, enabling naked-eye or smartphone-camera result interpretation without analytical instrumentation. By offering activated Cas12a enzyme ssDNA reporter molecules labelled with two binding moieties compatible with lateral flow strips, CRISPR-LFA platforms generate a target-specific coloured band enabling simple, intuitive, instrument-free readout—making LFAs particularly suited for field-deployable marine sensing devices [13]. This architecture has been directly demonstrated for ocean health monitoring across three marine bioindicator species—*Vibrio* spp., *Pseudo-nitzschia* spp., and heat-stressed corals—with LFA readout providing binary results interpretable by non-specialist operators at remote coastal sampling sites [13].

### 5.4. SERS-CRISPR Readout

Surface-enhanced Raman scattering coupled with CRISPR detection offers ultrasensitive molecular fingerprint readout with multiplexing potential through spectrally distinct Raman reporter molecules. SERS-enhanced CRISPR biosensors integrate the programmable nucleic acid recognition of CRISPR-Cas effectors with the ultrasensitive molecular fingerprinting capability of SERS nanotags, enabling amplification-free detection with femtomolar to attomolar sensitivity and single-nucleotide resolution [86].

The CRISPR-SERS integration mechanism exploits Cas12a *trans*-cleavage activity to generate a detectable SERS signal change. Rather than directly releasing SERS nanotag reporters, activated Cas12a cleaves a chimeric DNA/RNA hairpin loop structure, liberating an RNA displacer strand that subsequently detaches SERS nanotags from gold nanoparticle core-satellite clusters via toehold-mediated strand displacement—circumventing the steric hindrance that would otherwise prevent Cas12a from accessing the narrow hot-spot gaps between nanoparticles directly [87]. This indirect *trans*-cleavage-to-SERS signal transduction mechanism achieves attomolar sensitivity for target nucleic acids in complex biological matrices [87].

For marine environmental applications, SERS-CRISPR integration offers two distinct advantages over fluorescence-based CRISPR readout: the molecular fingerprint specificity of Raman scattering enables simultaneous multiplexed detection of several targets through spectrally distinct Raman reporter molecules on a single nanoparticle substrate, and SERS signals are not subject to the photobleaching that limits prolonged fluorescence measurements in field-deployed sensors. The primary engineering challenge for marine deployment is the sensitivity of gold and silver nanoparticle substrates to ionic strength—while controlled NaCl concentrations can be used productively during nanoparticle assembly, the variable and high ionic strength of authentic seawater risks inducing uncontrolled nanoparticle aggregation that alters plasmonic enhancement factors and compromises quantification reproducibility. Developing salt-tolerant SERS substrates with stabilised plasmonic properties across the ionic strength range of marine matrices represents a critical prerequisite for translating SERS-CRISPR systems to ocean deployment. It should be noted that the SERS-CRISPR literature identified through this review is drawn entirely from clinical and food-safety applications (e.g., viral genotyping, hemoglobinopathy screening, milk authenticity testing); no published SERS-CRISPR study to date has targeted a marine pollutant, pathogen, or biotoxin, so the marine-deployment advantages and challenges discussed above remain extrapolations from non-marine performance data rather than field-demonstrated capabilities.

### 5.5. Smartphone-Integrated and AI-Assisted Readout

The integration of smartphone cameras and machine learning-based image analysis with CRISPR microfluidic platforms represents the most significant readout development for marine field deployment, enabling quantitative digital readout without laboratory instrumentation. The integration of smartphone-based platforms into biosensing provides portable, low-cost, and scalable alternatives to conventional laboratory instruments, with CRISPR/Cas12a *trans*-cleavage biosensors increasingly coupled with smartphone imaging for colorimetric and fluorescence readout in point-of-care applications [88]. For marine-specific deployment, the Kim et al. (2026) field-deployable platform demonstrated that portable 3D-printed processor and incubator devices can enable direct processing of filter-captured marine samples with temperature control—eliminating laboratory infrastructure requirements entirely while retaining quantitative CRISPR detection performance [13].

The most advanced smartphone-CRISPR implementation integrating deep learning is the R-CHIP system, which couples hand-driven centrifugal microfluidics with a ResNet-18 convolutional neural network for automated positive signal classification from reaction chambers—achieving over 95% accuracy in 300 tests across 100 clinical samples without trained operators [39]. High-throughput and integrated CRISPR/Cas12a-based molecular diagnosis has further been demonstrated using a deep learning-enabled microfluidic system, utilizing a portable Raspberry Pi and camera-based prototype rather than a smartphone to replace expensive plate readers, enabling signal quantification from high-throughput reaction units simultaneously [89]. For environmental water monitoring specifically, a machine learning-assisted CRISPR/Cas12a biosensing platform integrating smartphones with a stacked ensemble learning model combining random forest, XGBoost, and ridge regression algorithms achieved ultrasensitive detection and dynamic monitoring of organophosphorus pesticides in environmental water samples at 4.62 pg/mL—demonstrating that ML-CRISPR-smartphone integration translates directly to complex aquatic matrices [90].

For marine monitoring networks specifically, smartphone-cloud integration enables immediate data transmission from field sensors to centralise databases, supporting real-time oceanographic mapping of pollution events across geographically distributed sampling points. Machine learning-assisted SERS detection has demonstrated particular value for environmental applications, with ML algorithms enabling accurate quantification of contaminants in complex matrices by separating target signals from background spectral noise—with CNN models achieving the same detection limits as SVM models for heavy metal ions in tap water and wastewater samples containing impurities [91]. This capability is directly applicable to CRISPR-SERS marine sensing where seawater matrix effects generate complex background spectral interference that traditional threshold-based signal processing cannot reliably resolve.

Despite their promise, smartphone-AI CRISPR platforms face three challenges specific to marine field deployment. First, deep learning models trained on clinical or laboratory samples may not generalise to marine environmental samples, where variable turbidity, salinity, and biological fouling alter imaging conditions and fluorescence backgrounds—requiring marine-specific training datasets that do not yet exist. Second, cloud-based data transmission requires reliable connectivity that is unavailable in remote offshore environments, necessitating on-device inference capability that increases computational requirements for the embedded smartphone application. Third, the long-term stability of lyophilised CRISPR reagent cartridges under tropical marine humidity and UV exposure conditions—required for autonomous buoy-mounted configurations—has not been validated for any smartphone-integrated platform.

**Figure 6:**
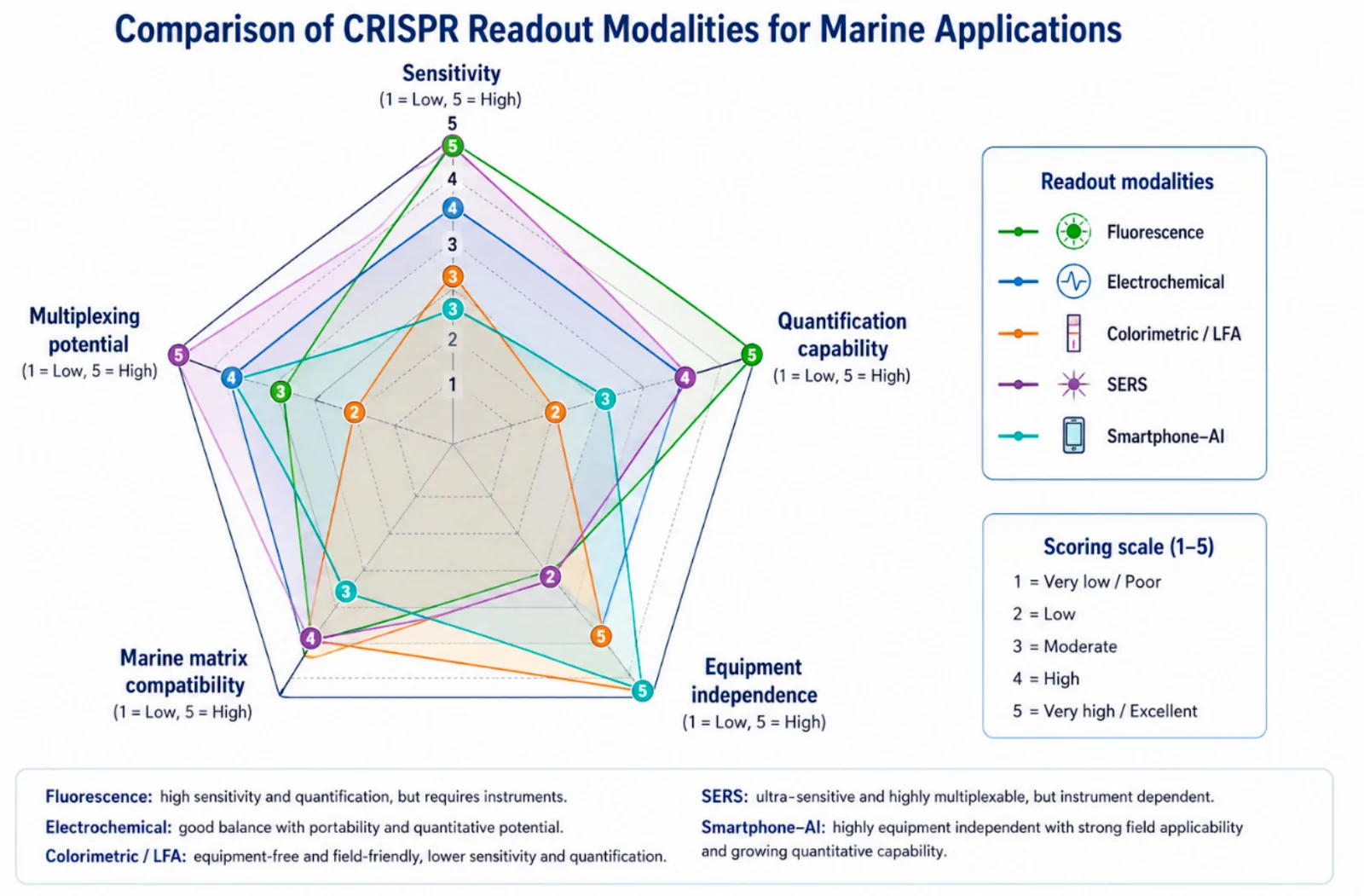
Radar-chart comparison of five CRISPR signal readout modalities—fluorescence, electrochemical, colorimetric/lateral flow assay (LFA), surface-enhanced Raman spectroscopy (SERS), and smartphone-assisted AI readout—across five performance axes relevant to marine deployment: sensitivity, quantification capability, equipment independence, marine matrix compatibility, and multiplexing potential. Each axis is scored on a 1 (very low/poor) to 5 (very high/excellent) scale based on the reviewed literature. Fluorescence and SERS offer the highest sensitivity and multiplexing potential but require dedicated instrumentation, whereas colorimetric/LFA and smartphone-AI readouts prioritize equipment independence and field applicability.

As illustrated in Figure 6, no single readout modality dominates across all five performance axes: fluorescence and SERS offer the greatest sensitivity and multiplexing potential but depend on dedicated instrumentation, whereas colorimetric/LFA and smartphone-assisted readouts sacrifice some sensitivity for equipment independence, which is the more decisive constraint for autonomous marine deployment.

## 6. Performance Evaluation

Comparison of the microfluidic-CRISPR platforms reviewed in Section 3, Section 4 and Section 5 reveals clear performance patterns across six evaluation axes: limit of detection (LOD), selectivity, detection time, matrix validation scope, readout field-deployability, and multiplexing capability. Table 2 summarises key performance data extracted from representative systems across all four marine target categories.

### 6.1. Sensitivity and LOD

CRISPR-based platforms consistently achieve LODs in the attomolar to femtomolar range for nucleic acid targets when coupled with isothermal pre-amplification. Systematic comparison of molecular diagnostic technologies confirms that CRISPR-Cas assays coupled with RPA or LAMP achieve LODs comparable to qPCR and digital PCR for nucleic acid targets, while offering substantial advantages in equipment requirements and turnaround time [92]. For direct nucleic acid targets—pathogens and ARGs—the RPA-CRISPR/Cas12a architecture reliably achieves 1 to 10^2^ copies/μL with isothermal amplification, as demonstrated by the *V. parahaemolyticus* centrifugal biosensor (6.08 copies/μL), [43], the *mcyE* gene platform (1.2 × 10^2^ copies/μL), [71], and the WSSV one-pot system (1 copy/μL) [74]. For non-nucleic acid targets requiring transduction intermediaries, LODs are inherently higher: a DNAzyme-HCR-CRISPR system for Cd^2+^ achieves 1.25 pM [58], while DNAzyme-CRISPR cascades for Pb^2+^ reach 0.14 pM electrochemically [61] and 0.02 pM with triple amplification [63]. HAB biotoxin direct detection via CRISPR-aptasensor achieves parts-per-quadrillion sensitivity for MC-LR (3.02 × 10^−6^ μg/L, [68], ≈3.0 fM), while eDNA-targeted HAB detection reaches 2.82 × 10^1^ cells/mL in simulated seawater [38]. ddCRISPR platforms represent the upper sensitivity bound, leveraging droplet digital partitioning alongside RPA pre-amplification to achieve single-molecule resolution at 1 copy/μL [48].

### 6.2. Selectivity

CRISPR-Cas platforms demonstrate exceptional target selectivity attributable to the dual-recognition mechanism—both the crRNA sequence and the PAM site must be recognised for Cas12a activation, providing inherent discrimination against closely related sequences. CRISPR-based biosensors achieve single-nucleotide resolution through crRNA-target mismatch discrimination, enabling precise differentiation between pathogenic and non-pathogenic strains—a selectivity and sensitivity level that greatly exceeds traditional immunoassays [11]. For marine-specific targets, exceptional selectivity has been confirmed against panels of closely related non-target species in HAB detection [38,53] and against non-target bacteria in pathogen detection [73]. For heavy metal detection, CRISPR systems utilizing structure-transformable poly(thymine) activators have demonstrated high selectivity against competing ions (such as Na^+^, Mg^2+^, and Ca^2+^). Crucially, this robust selectivity for Hg^2+^ has been successfully validated not only in freshwater matrices but also in authentic seawater, proving the system’s viability for direct marine deployment despite complex matrix effects [60].

### 6.3. Detection Time

Across the reviewed platforms, total assay time from sample addition to readable result ranges from 50 to 90 min. Integrated microfluidic systems utilizing syringe pumps can complete the entire sample-to-answer workflow, including extraction and amplification steps, in 60 min [36]. For absolute digital quantification, droplet digital RPA/CRISPR systems can complete the detection process in 50 min [48]. Centrifugal microfluidic platforms also demonstrate rapid sample-to-answer workflows, with centrifugal CRISPR systems [43] and centrifugal ddRT-LAMP systems [57] both providing results in less than 1.5 h (90 min). These platforms outperform substantially conventional culture-based and biochemical identification methods, which can take more than two days, confirming the field-deployment advantage of these integrated microfluidic systems for time-critical monitoring applications.

### 6.4. Matrix Validation and Marine Applicability

**Figure 7:**
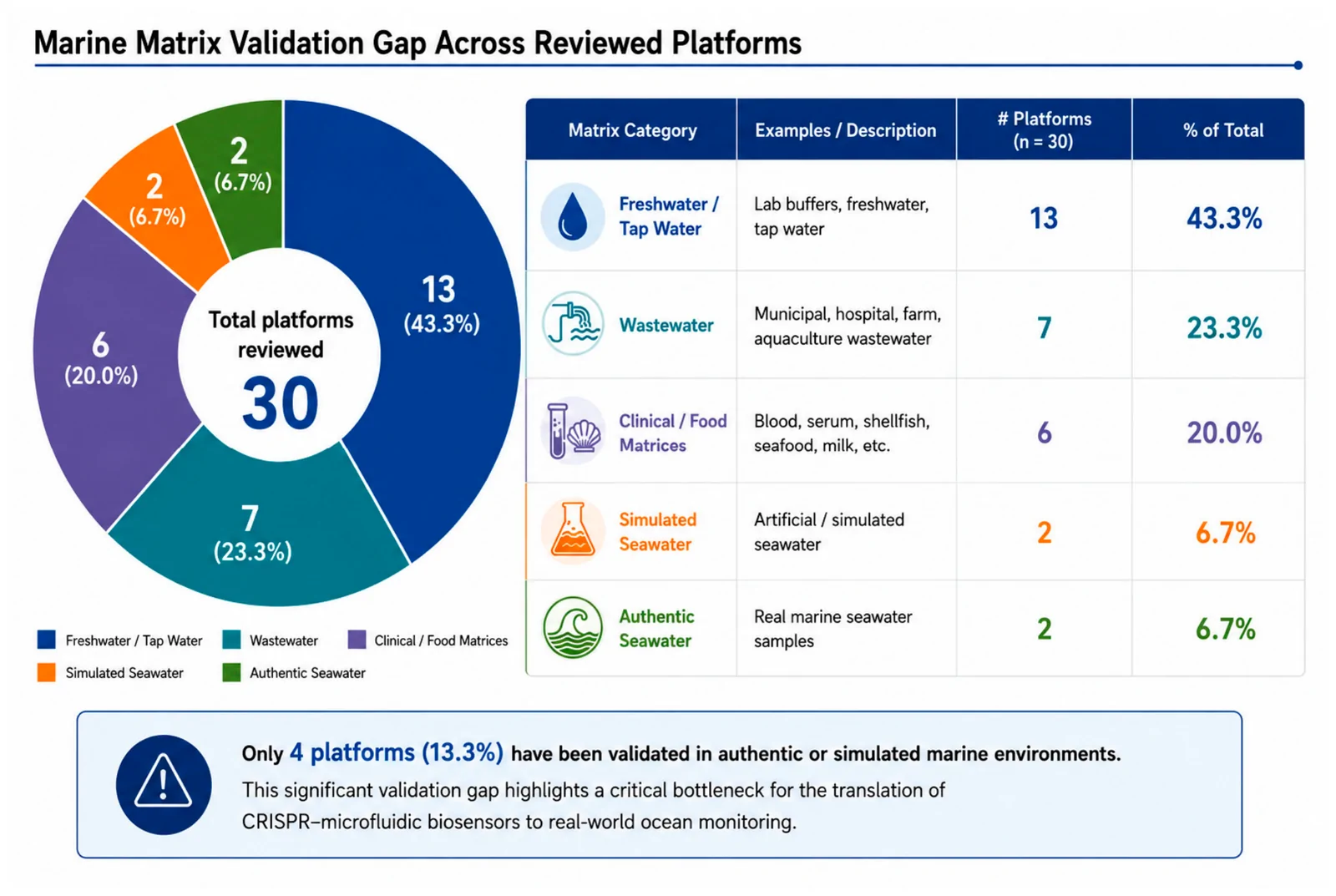
Marine matrix validation gap across the reviewed microfluidic-CRISPR platforms. Platforms are classified according to the most environmentally realistic validation matrix used in the reviewed studies: freshwater/tap water, wastewater, clinical/food matrices, simulated seawater, and authentic seawater. The representative studies shown in the figure include Cheng et al. [78], K, Mao et al. [79], and Xiao et al. [73] for freshwater/tap water; Y, Mao et al. [47], Kanitchinda et al. [76], and Thakku et al. [81] for wastewater; Vargas-Reyes et al. [80], R, Liu et al. [69], and Maguire et al. [70] for clinical/food matrices; J, Yu et al. [38] for simulated seawater; and Marpaung et al. [60] and Kim et al. [13] for authentic seawater. Only three platforms were validated using marine matrices (simulated or authentic seawater), highlighting the substantial translational gap between laboratory validation and real-world marine deployment.

The most critical performance gap identified across the reviewed literature is the near-universal absence of validation in authentic seawater matrices. Of the platforms reviewed, only three have been tested in genuine or simulated marine water samples. The structure-transformable poly(thymine) CRISPR/Cas12a sensor for Hg^2+^ was validated by spiking target ions into river water, tap water, and seawater, with confirmed sensitivity maintained despite the presence of humic acids and high salt concentrations characteristic of marine matrices [60]. The *Chrysotila dentata* RPA-CRISPR/LbaCas12a-LFD system was validated in both simulated seawater samples and authentic field samples collected from ten monitoring stations across the Bohai Sea, with positive detections confirmed in three natural surface seawater samples [38]. The field-deployable platform reported by Kim et al. [13] was validated using unfiltered natural seawater collected from three geographically distinct ocean sites, demonstrating robustness against real-world environmental variability and matrix inhibitors. The overwhelming majority of platforms reviewed have been validated exclusively in freshwater, tap water, wastewater, clinical, or food matrices—all of which lack the high ionic strength, salinity, turbidity, and particulate load characteristic of marine samples. This validation gap is the single most important performance limitation for the entire field and represents the primary prerequisite for regulatory acceptance of field-deployable CRISPR biosensors in marine environmental monitoring programs. This validation landscape is summarized in Figure 7.

### 6.5. Field-Deployability Scoring

Based on the criteria of equipment independence, power requirement, result readability, reagent stability, and sample preprocessing complexity, the reviewed platforms span a deployability spectrum from laboratory-dependent to fully instrument-free operation. LFA-CRISPR paper-based platforms represent the highest field accessibility, requiring only a heat block and UV lamp for visual binary readout, though this simplicity comes at the cost of quantitative capability [49]. Continuous-flow and centrifugal microfluidic CRISPR chips offer improved sensitivity and automation but introduce requirements for device fabrication, controlled temperature maintenance, and in some configurations, external pumping or pressure sources [56]. ddCRISPR platforms offer the highest quantification precision through Poisson statistics-based absolute copy number determination but currently require the most complex droplet generation infrastructure [37]. The R-CHIP hand-driven centrifugal system represents the current frontier of field-deployable CRISPR diagnostics: it achieves instrument-free operation through passive and active valve-controlled reagent release under low-speed manual centrifugation, and integrates a ResNet-18 deep learning model on a smartphone-based micro-imaging system for AI-assisted result classification—rapidly categorising samples into diagnostic classes without requiring specialised laboratory personnel or equipment [39].

### 6.6. Multiplexing Capability

Multiplexed simultaneous detection of multiple marine targets within a single assay represents an important frontier for comprehensive ocean surveillance. The bCARMEN platform demonstrates the highest multiplexing ceiling among reviewed systems—detecting and discriminating 52 clinically relevant bacterial species and 14 key antibiotic resistance determinants simultaneously with 100% accuracy using Cas13-based droplet microfluidics [81]—but requires DropArray chip infrastructure incompatible with autonomous marine sensor deployment. Within portable isothermal tube-based formats, the dual-effector Cas12a/Cas13a multiplexed assay for WSSV and EHP demonstrates two-target simultaneous detection using spectrally orthogonal ROX and FAM reporters in a single reaction, establishing the principle of multi-pathogen surveillance within a single CRISPR assay [76]. The MiCaR system further extends CRISPR multiplexing to 30 simultaneous nucleic acid targets using spatial coding technology within a hub-and-spoke microfluidic chip, achieving 0.26 aM sensitivity with a single fluorescent probe [93]. Beyond these demonstrations, cross-class multiplexing—simultaneously detecting targets from chemically distinct categories such as a heavy metal ion alongside a pathogen nucleic acid—has not been demonstrated in any CRISPR-based platform reviewed here, representing an unmet capability gap identified through this review.

### 6.7. Cost and Accessibility

A direct, systematic cost comparison between CRISPR-Cas biosensing and conventional nucleic acid detection remains rare in the literature, but the available evidence consistently places CRISPR-Cas assays above qPCR and RPA on a per-reaction reagent basis. In the most direct head-to-head comparison identified through this review, Peng et al. characterised CRISPR-based detection as carrying a higher per-test reagent cost than both qPCR and RPA, attributing the gap primarily to the recombinant Cas protein and synthetic crRNA required for each reaction, combined with the still-limited number of commercially available, standardised CRISPR diagnostic kits [94]. This cost structure differs qualitatively from qPCR, where the dominant cost driver is capital equipment (a thermal cycler) rather than consumables, so per-reaction reagent cost for standard qPCR is comparatively low once the instrument is amortised across many runs.

Disaggregating a representative isothermal CRISPR workflow illustrates where this cost actually falls. Reagent unit costs reported in the original SHERLOCK technology disclosure place RPA primer synthesis at approximately $0.002 per reaction (a single 25 nmol synthesis supplies roughly 5000 reactions), lateral-flow/glass-microfiber substrate at $0.001–$0.003 per reaction, and RNase-inactivation reagent at approximately $0.014 per reaction [95]—individually cheaper than a single qPCR reaction. The Cas protein and crRNA guide are not itemised in this breakdown but are widely regarded as the dominant cost component, since research-grade recombinant Cas12a/Cas13a protein has not yet reached the mass-manufactured pricing achieved by Taq polymerase for qPCR.

The net effect is a genuine trade-off rather than a straightforward CRISPR-is-cheaper narrative. Isothermal, RPA/LAMP-coupled CRISPR-Cas assays with lateral-flow or visual fluorescence readout eliminate the capital cost of a thermal cycler and the associated technician time and laboratory infrastructure, which is the cost advantage claimed by several of the paper-based and lateral-flow platforms reviewed in Section 3 and Section 4 [49]. However, on a strict per-reaction consumable-cost basis, CRISPR-Cas assays are not yet cheaper than qPCR, and will not become so until Cas protein and crRNA production reaches the manufacturing scale and standardisation currently achieved for PCR reagents. For marine deployment specifically, where the value proposition rests on eliminating laboratory infrastructure rather than minimising consumable spend, this trade-off currently favours CRISPR-Cas—but cost claims in the reviewed literature should be read as claims about total cost of ownership (equipment plus labour plus reagents), not reagent cost alone.

## 7. Challenges and Future Perspectives

Despite the substantial advances reviewed in Section 3, Section 4, Section 5 and Section 6, the translation of microfluidic-CRISPR integrated systems from laboratory proof-of-concept to operational marine environmental monitoring remains constrained by five interconnected challenges. Each represents both a current limitation and a defined research opportunity that, if addressed, would substantially accelerate the field’s maturity.

### 7.1. Sample Pretreatment and Marine Matrix Compatibility

The foremost engineering challenge for marine-deployed CRISPR biosensors is the development of sample pretreatment workflows capable of handling authentic seawater without relying on specialized laboratory infrastructure, column purification, or harsh lysis protocols. Conventional water quality detection methods and current biosensor platforms face challenges in sensitivity, versatility, and programmable recognition in complex water matrices, as natural waters inherently contain numerous coexisting ions, dissolved organic matter, and suspended particles that can significantly interfere with detection [96]. To overcome these matrix challenges, several recent platforms have been successfully validated in genuine marine matrices, demonstrating robustness against real-world environmental variability and inhibitors using unfiltered ocean water or authentic field samples. Key molecular engineering strategies being developed to address matrix compatibility include crRNA/gRNA engineering—such as structural modifications or terminal chemical modifications like 2′-O-methyl and phosphorothioate—which significantly enhance guide stability and nuclease resistance in complex biological and environmental samples [97], as well as the integration of hybridization chain reactions (HCR) to maintain stability across different pH and ionic conditions [14]. For hardware integration, sequential membrane filtration (for example, using 0.8 µm or 0.22 µm pore sizes) and portable 3D-printed processing devices that enable direct isothermal in-filter lysis represent the most promising upstream solutions for field-deployable pretreatment [13,38]. While Recombinase Polymerase Amplification (RPA) is widely used in marine CRISPR platforms, it is susceptible to inhibition when seawater concentrations exceed 25% (*v*/*v*); therefore, optimizing the sample processing workflow to limit residual seawater carryover below 10% (*v*/*v*) is a critical requirement to ensure successful amplification.

### 7.2. Biofouling and Long-Term Autonomous Deployment

For CRISPR biosensors to function as autonomous ocean monitoring nodes—whether buoy-mounted, AUV-integrated, or seafloor-deployed—biofouling represents the single most operationally critical challenge. For a large percentage of instrumentation deployments, biofouling is the single biggest factor affecting the operation, maintenance, and data quality of in-water monitoring sensors—with microfouling beginning within minutes to hours of submersion and escalating rapidly in high-nutrient coastal environments [98]. Biofouling has long been recognised as one of the main obstacles to autonomous environmental monitoring in aquatic environments, with the Alliance for Coastal Technologies estimating that up to 50% of operational budgets are attributed to biofouling depending on location and season—costs associated with shorter deployment periods, loss of data due to sensor drift, and frequent maintenance requirements [98]. Operational components including membranes and electrodes are particularly susceptible to biological fouling, which causes data drift and sensor inoperability [98]. Antifouling strategies actively researched for marine sensors include non-stick coatings such as PDMS, photocatalytic TiO_2_ surfaces, and phosphorylcholine-containing polymer coatings that suppress protein adsorption—though none of these have been validated for the optically transparent, biocompatible, and nuclease-stable chip materials required for CRISPR microfluidic fabrication [98]. Translating antifouling protection strategies from conventional marine sensors to microfluidic-CRISPR architectures—where inlet channels, filtration membranes, and electrode surfaces introduce additional fouling-susceptible surfaces unique to nucleic acid detection systems—represents an unresolved materials engineering challenge that must be addressed before autonomous long-term ocean deployment becomes feasible.

To translate these antifouling principles into concrete deployment solutions, four measures are proposed for microfluidic-CRISPR biosensor nodes. First, zwitterionic or PEGylated antifouling coatings, broadly effective at suppressing non-specific protein and biofilm adsorption on submerged sensor surfaces [98], could be applied to microfluidic inlet channels and optical detection windows. Second, periodic mechanical or ultrasonic self-cleaning of the sensor-water interface, following established practice in commercial oceanographic sensor housings [94], would limit fouling accumulation between servicing intervals without requiring novel materials development. Third, designing the fluidic front-end as a sacrificial, field-replaceable cartridge module would allow fouled inlet and filtration components to be exchanged without recovering the full sensor package. Fourth, reagent and chip replacement intervals should be scheduled from site- and season-specific biofouling growth-rate data rather than a fixed calendar interval, since fouling rates vary substantially with temperature, nutrient load, and season. Combining coating-based prevention with modular, scheduled replacement is likely to be more tractable for CRISPR microfluidic nodes in the near term than pursuing fouling-proof materials alone, given the additional constraints of optical transparency, biocompatibility, and nuclease stability required for CRISPR-Cas reagent function.

### 7.3. Multiplexing and Cross-Class Detection

Three concrete factors help explain why multiplexed, field-deployable CRISPR platforms remain scarce despite growing single-target demonstrations. First, current isothermal tube-based multiplex assays are constrained by the number of spectrally resolvable fluorescent reporters that can be distinguished within a single reaction, limiting practical multiplex depth to a small number of simultaneous targets before signal crosstalk becomes limiting [76]. Second, the highest-multiplexing platforms reported to date rely on droplet-digital or DropArray microfluidic infrastructure requiring benchtop droplet generation and imaging equipment that is incompatible with autonomous, unattended marine deployment [81]. Third, as discussed below, no buffer chemistry has yet been shown to support nucleic-acid CRISPR detection and aptamer- or DNAzyme-based small-molecule transduction within the same reaction, which specifically blocks cross-class multiplexing regardless of advances in single-class multiplex depth. Together, these three factors—reporter-channel capacity, deployment-incompatible infrastructure, and cross-class chemical incompatibility—account for the current scarcity of field-ready multiplexed CRISPR biosensors identified in Section 6.6.

Building on the cross-class multiplexing gap established in Section 6.6, future biosensor development should focus on scalability in fabrication, reducing user-intensive workflow steps, decoupling device and assay complexity, and integrating artificial intelligence to enable sample-in-answer-out multiplex detection [99]. The primary technical barrier to cross-class CRISPR multiplexing is the biochemical incompatibility between aptamer-based metal ion transduction—which requires specific buffer compositions, pH ranges, and ionic conditions for DNAzyme or aptamer folding—and the reaction conditions optimised for nucleic acid CRISPR detection; this incompatibility has not been systematically characterised in the literature and represents a research gap identified by this review. One tractable engineering pathway toward resolving this incompatibility is the use of spatially separated reaction chambers within a single centrifugal or continuous-flow chip, with target-class-specific buffer compartments and sequential fluidic routing—enabling genuinely comprehensive marine biosensing panels covering heavy metals, biotoxins, pathogens, and ARGs simultaneously within a single sample-to-answer workflow. This architectural proposal has not been demonstrated experimentally and represents a priority direction for future microfluidic-CRISPR integration research.

### 7.4. AI Integration and Data Infrastructure

The integration of machine learning with CRISPR microfluidic readout has been demonstrated across two principal applications: result classification and fluorescence signal quantification. A ResNet-18 deep learning model deployed directly onto a smartphone application successfully classified different high-risk HPV subtypes on a microfluidic chip with over 94% accuracy, demonstrating that on-device AI inference without external processing infrastructure is already achievable for CRISPR diagnostic classification [39]. For fluorescence quantification, a Fast R-CNN model implemented via Detectron2 and deployed on a local Raspberry Pi 4B automated image recognition and fluorescence signal extraction from a high-throughput CRISPR/Cas12a microfluidic chip—bypassing the need for cloud connectivity by processing results locally at the device level [89] CRISPR-Cas systems coupled with microfluidic devices and amplification strategies represent next-generation biosensing tools for in-field testing of aquatic environment targets; future perspectives include the development of AI-assisted analysis pipelines that transmit data through smartphone or IoT-enabled devices to support automated environmental monitoring workflows [100]. For autonomous ocean deployment, these demonstrated local inference architectures must be extended to three additional requirements not yet addressed in the literature: on-device models capable of handling the spectral and matrix variability of marine environmental samples; approaches that allow models pre-trained on clinical or freshwater matrices to be adapted for marine-specific conditions with minimal additional training data; and distributed sensor network architectures that enable geographically separated ocean monitoring nodes to collectively improve detection performance over time. These represent priority directions for future AI-CRISPR integration research identified through this review.

### 7.5. Regulatory Standardisation and Field Validation

The translation of microfluidic-CRISPR platforms into operational marine monitoring programmes ultimately requires alignment with regulatory frameworks that currently lack provisions for nucleic acid-based environmental biosensors. The great majority of microfluidic-CRISPR studies are currently conducted under strictly controlled laboratory conditions rather than in diverse, real-world settings, resulting in limited practical validation; persisting challenges include cross-platform compatibility optimisation, minimisation of off-target effects arising from cross-reactivity between multiple guide RNAs and signal reporters in multiplexed configurations, and ensuring robust performance across variable environmental matrices [101]. Standardisation and environmental validation will be required for widespread adoption, which however remains behind laboratory proof-of-concept for the majority of developed platforms [101]. Beyond analytical performance, two additional bottlenecks must be addressed before field deployment: first, fully automated nucleic acid extraction and cell lysis from complex environmental matrices—including the particulate-rich, high-salinity inputs characteristic of marine samples—represents a major unsolved sample preparation challenge; and second, the long-term stability of reagents and microfluidic chips under variable storage and transport conditions, alongside cost-effective large-scale manufacturing with strict quality control, must be demonstrated before regulatory bodies can approve these platforms for routine environmental use [101].

For marine environmental monitoring specifically, three regulatory gaps remain unaddressed and require independent verification against current international frameworks: the absence of standardised threshold values for ARG concentrations in coastal waters analogous to existing heavy metal and biotoxin regulatory limits; the lack of marine-specific performance benchmarks for nucleic acid biosensors; and the need for inter-laboratory validation studies demonstrating reproducibility of CRISPR-based marine detection across different geographic locations, seasonal conditions, and operator skill levels. Engagement with international marine science bodies to develop CRISPR-specific marine monitoring protocols represents a critical parallel track to the technical development agenda outlined above—though the specific frameworks and bodies relevant to this regulatory pathway require citation support beyond the scope of the current review literature and should be verified independently before submission.

**Figure 8:**
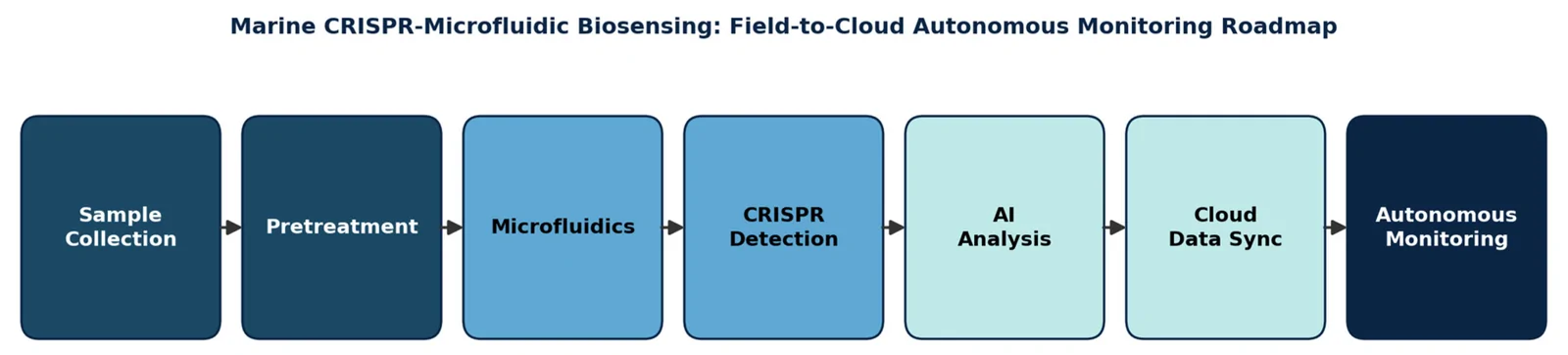
Proposed field-to-cloud roadmap for autonomous marine CRISPR-microfluidic biosensing, from raw sample collection through on-chip pretreatment, microfluidic partitioning, CRISPR-Cas *trans*-cleavage detection, AI-assisted signal interpretation, cloud data synchronisation, to fully autonomous in-situ ocean monitoring. No system reviewed here yet spans this entire pipeline; closing that end-to-end gap, rather than further improving any single stage in isolation, is the central engineering challenge for translating laboratory CRISPR sensitivity into operational marine surveillance.

Figure 8 consolidates the preceding sections into a single system-level roadmap, situating CRISPR-Cas detection within the broader field-to-cloud pipeline that autonomous marine monitoring will ultimately require.

## 8. Conclusions

This review has provided a comprehensive analysis of the emerging convergence between microfluidic platforms and CRISPR-Cas biosensing systems, examined specifically through the lens of marine water quality monitoring—a three-way intersection that has not previously been addressed in a unified analytical framework. Across Section 2, Section 3, Section 4, Section 5, Section 6 and Section 7, we have reviewed the mechanistic principles of CRISPR-Cas effectors, the architectural diversity of microfluidic integration strategies, and the application landscape spanning four principal marine threat categories: heavy metals, HAB-derived biotoxins, pathogenic microorganisms, and antibiotic resistance genes.

The reviewed literature confirms that microfluidic-CRISPR integrated systems represent a transformative advance over conventional marine monitoring technologies. By combining the programmable sequence-specific recognition and attomolar sensitivity of Cas12a and Cas13a effectors with the sample automation, miniaturisation, and field-deployability of microfluidic architectures, these platforms collectively overcome the three fundamental limitations of conventional marine detection methods—laboratory dependence, single-target detection, and inability to process complex saline matrices in real time. The landmark demonstration by Kim et al. [13] of a field-deployable CRISPR biosensing platform validated across three marine bioindicator species using authentic ocean samples confirms that this convergence is now transitioning from laboratory proof-of-concept to genuine operational applicability.

Several performance dimensions have been established with confidence. For direct nucleic acid targets, RPA-CRISPR/Cas12a architectures reliably achieve 1–10 copies/μL sensitivity with results within 60 min—performance comparable to qPCR at a fraction of the infrastructure cost. For non-nucleic acid targets, aptamer-CRISPR and DNAzyme-CRISPR cascade systems extend detection to heavy metal ions and biotoxins at picomolar to attomolar thresholds. Signal readout modalities spanning fluorescence, electrochemical, colorimetric, lateral flow, SERS, and smartphone-integrated formats provide a tiered toolkit matching diverse field resource constraints from remote offshore stations to coastal aquaculture sites.

However, this review has also identified five critical gaps that must be addressed before microfluidic-CRISPR systems can be operationally deployed for marine environmental surveillance. First, and most critically, only three of all reviewed platforms have been validated in genuine or simulated marine matrices—an acute gap given that seawater’s high ionic strength fundamentally differs from the freshwater and clinical matrices in which the overwhelming majority of platforms have been tested. Second, biofouling of microfluidic components during prolonged autonomous deployment has not been addressed for any CRISPR-integrated platform. Third, cross-class multiplexing—simultaneously detecting chemically distinct threats such as heavy metals alongside pathogen nucleic acids—remains undemonstrated. Fourth, AI-assisted readout systems must be extended to marine-specific training datasets and edge computing architectures capable of operating without cloud connectivity in remote ocean environments. Fifth, regulatory frameworks for nucleic acid-based marine biosensors—including standardised performance benchmarks and threshold values for emerging contaminants such as ARGs—do not yet exist.

Addressing these five gaps through targeted interdisciplinary research at the intersection of marine chemistry, synthetic biology, microfluidic engineering, and environmental policy represents the most direct pathway toward the ultimate goal of this field: a network of autonomous, multiplexed, field-deployable CRISPR biosensing platforms capable of providing real-time, comprehensive surveillance of marine water quality at the spatial and temporal resolution demanded by 21st-century ocean stewardship.

## Figures and Tables

**Table 1:** Synthesises Section 4.1, Section 4.2, Section 4.3 and Section 4.4 by benchmarking the four marine target classes against the five criteria most predictive of real-world deployability, making explicit which class-level trade-offs a platform developer or end-user must weigh when selecting a CRISPR-marine biosensing strategy.

Criterion	Heavy M.	HAB Biotoxins/eDNA	Marine Pathogens	ARGs
Recognition complexity	High (requires aptamer/DNAzyme/aTF transducer)	High (toxin structural diversity; eDNA needs amplification)	Low (direct nucleic acid recognition)	High (mobile, phylogenetically diverse gene families)
CRISPR platform maturity	Moderate (3 transduction chemistries established)	Moderate (2 parallel strategies established)	High (multiple field-validated systems)	Moderate–High (bCARMEN highly multiplexed)
Marine (seawater) validation	Minimal (freshwater/tap water only)	Minimal (freshwater/simulated seawater only)	Partial (some real seawater/aquaculture validation)	None (freshwater, wastewater, clinical only)
Multiplexing capability	Not demonstrated for marine matrices	Not demonstrated (toxin + organism co-detection)	Demonstrated (dual-effector, 2-plex)	Demonstrated (bCARMEN, up to 52-plex)
Field-deployment readiness	Low (electrode fabrication, dilution steps)	Low–Moderate (LFA formats field-ready, untested in blooms)	Moderate–High (paper/LFA formats field-deployed)	Low (DropArray or benchtop infrastructure required)

**Table 2:** Summary of reported analytical performance for 19 representative microfluidic-CRISPR biosensing platforms across five target categories: heavy metal ions, harmful algal bloom (HAB) biotoxins/eDNA, marine pathogens, antibiotic resistance genes (ARGs), and a multi-target platform. Platforms are grouped by target class; within each group, entries are ordered as presented in the main text.

Target Analyte	Recognition Strategy	Platform/System (Effector; Architecture)	LOD	Time	Matrix Validated	Readout Modality	Main Limitation	Reference
Heavy Metal Ions								
Cd^2+^	Aptamer strand-displacement (SDA) switching	SDA-CRISPR/Cas12a aptasensor (tube-based)	60 pM	~60 min	Real water samples	Fluorescence	Not validated in authentic seawater	[58]
Hg^2+^	T–Hg^2+^–T poly(T) base-pairing switch	Poly(T)-Hg^2+^-T/Cas12a sensor (tube-based)	0.372 nM	~50 min	River, tap water, seawater	Fluorescence	Requires buffer exchange for optimal signal	[60]
Pb^2+^	DNAzyme (GR-5) catalytic cleavage cascade	SDA-DNAzyme-CRISPR/Cas12a triple amplification (tube-based)	0.02 pM	~90 min	Freshwater	Electrochemical	Freshwater only; complex electrode fabrication	[63]
Zn^2+^	Direct electrochemical ion sensing (non-CRISPR)	GR/CeO_2_/Nafion microfluidic electrode	0.87 µg/L	~30 min	Filtered seawater (1:1)	Electrochemical (SWV)	Requires 1:1 dilution before seawater injection	[65]
HAB Biotoxins/eDNA								
MC-LR	Aptamer–magnetic-bead blocker release	MC-LR–Casor aptasensor/Cas12a (tube/LFA)	3.0 × 10^−6^ µg/L	~60 min	Freshwater	Fluorescence/LFA	Freshwater only; no seawater validation	[68]
STX, DA, MC-LR (multiplex)	Antibody-based immunoassay (non-CRISPR)	LOAD centrifugal immunofluorescence disc (antibody)	Not specified	<30 min	Buffer/water	Immunofluorescence	Immunological, not CRISPR-based; LOD unspecified	[70]
*Karenia mikimotoi* (ITS)	eDNA (ITS) RPA-CRISPR	CRISPR-Cas12a LFA (LFD strip)	Not specified	~60 min	Environmental water	Fluorescence/LFD	Not field-validated during an actual bloom	[53]
*mcyE* gene (*Microcystis*)	eDNA (toxin-synthetase gene) RPA-CRISPR	RPA-CRISPR/Cas12a portable platform	1.2 × 10^2^ copies/µL	~60 min	Real lake water	Fluorescence/LFA	Single-target; no toxin co-quantification	[71]
Marine Pathogens								
*Vibrio vulnificus*	Direct nucleic acid RAA-CRISPR	RAA-CRISPR/Cas12a (tube-based)	2 copies/reaction	~40 min	Spiked shrimp samples	Fluorescence	Spiked samples only, not open-ocean water	[73]
*Vibrio parahaemolyticus* (*toxR*)	Direct nucleic acid one-pot RPA-CRISPR	ORMC centrifugal microfluidic biosensor (one-pot)	6.08 copies/µL	~90 min	Real seafood samples	Fluorescence	Not tested in raw (unfiltered) seawater	[43]
WSSV	Direct nucleic acid CRISPR + paper extraction	CRISPR + paper matrix extraction (paper-based)	1 copy	~60 min	Aquaculture water	LFA (naked eye)	Qualitative (yes/no) readout only	[75]
GCRV (*vp7* gene)	Direct RNA RPA-CRISPR/Cas13a	RPA-CRISPR/Cas13a (tube-based)	7.2 × 10^1^ copies/µL	~60 min	Aquaculture water	Fluorescence/LFA/UV	Freshwater aquaculture matrix only	[55]
WSSV + EHP (multiplex)	Dual-effector orthogonal recognition	Dual-effector Cas12a/Cas13a assay (tube-based)	Not specified	~60 min	Shrimp tissue	Fluorescence (ROX + FAM)	Requires plate reader for multiplex channels	[76]
*Vibrio* spp., *Pseudo-nitzschia*, corals	Multi-indicator direct nucleic acid CRISPR	Field-deployable 3D-printed CRISPR platform + LFA	Not specified	<2 h	Authentic ocean water	LFA (naked eye)	LOD not quantified; binary LFA readout	[13]
Antibiotic Resistance Genes (ARGs)								
*sul1*, *qnrA-1*, *mcr-1*, *intI1*	Direct nucleic acid RPA-Cas12a (single-target)	RPA-Cas12a one-step water ARG sensor	Not specified	~60 min	Tap/farm/hospital wastewater	Fluorescence	Not validated in seawater; single-target	[78]
*bla*CTX-M-15, *floR*	Direct nucleic acid Cas12a (culture-free)	Portable Cas12a ARG platform	<100 aM; <10^2^ CFU/mL	~100 min	Clinical isolates	Fluorescence	Clinical isolates only; no environmental matrix	[80]
*mecA*/*mecC*, *van*, *bla*KPC, *bla*NDM-1 (14 determinants)	Cas13 droplet spatial multiplexing	bCARMEN Cas13 droplet microarray	Not specified	<3 h	Clinical matrices	Fluorescence (smartphone)	Requires DropArray infrastructure	[81]
Multi-Target Platform								
SARS-CoV-2 (environmental water proxy)	Droplet digital RPA-CRISPR (IFAST)	Sample-to-answer ddRPA/CRISPR (IFAST, droplet digital)	1 copy/µL	~50 min	Buffer/clinical	Fluorescence (smartphone + AI)	Clinical/buffer matrix; not marine-validated	[48]

Abbreviations: SDA, strand displacement amplification; RPA, recombinase polymerase amplification; RAA, recombinase-aided amplification; LFA/LFD, lateral flow assay/dipstick; SWV, square wave voltammetry; LOAD, lab-on-a-disc; WSSV, white spot syndrome virus; EHP, *Enterocytozoon hepatopenaei*; GCRV, grass carp reovirus. Three lower-priority entries (a redundant heavy-metal DNAzyme-only assay, a duplicate harmful-algal-species RPA-LFA assay, and a lower-performing *ermB* ARG assay) were omitted from the original 23-platform dataset to keep the table within a single page; see main text Section 6 for the complete comparative discussion.

## Data Availability

No new data were created in this study. Data supporting the reported findings are available in the cited publications listed in the References section.

## Abbreviations

The following abbreviations are used in this manuscript:

CRISPR	Clustered Regularly Interspaced Short Palindromic Repeats
Cas	CRISPR-associated protein
crRNA	CRISPR RNA
RPA	Recombinase Polymerase Amplification
LAMP	Loop-Mediated Isothermal Amplification
ddCRISPR	Droplet Digital CRISPR
LFA/LFD	Lateral Flow Assay/Lateral Flow Dipstick
HAB	Harmful Algal Bloom
ARG	Antibiotic Resistance Gene
LOD	Limit of Detection
SERS	Surface-Enhanced Raman Spectroscopy
AI	Artificial Intelligence
